# Frequency and signature of somatic variants in 1461 human brain exomes

**DOI:** 10.1038/s41436-018-0274-3

**Published:** 2018-09-14

**Authors:** Wei Wei, Michael J. Keogh, Juvid Aryaman, Zoe Golder, Peter J. Kullar, Ian Wilson, Kevin Talbot, Martin R. Turner, Chris-Anne McKenzie, Claire Troakes, Johannes Attems, Colin Smith, Safa Al Sarraj, Chris M. Morris, Olaf Ansorge, Nick S. Jones, James W. Ironside, Patrick F. Chinnery

**Affiliations:** 1 Department of Clinical Neurosciences, University of Cambridge, Cambridge Biomedical Campus, Cambridge, UK; 2 Department of Mathematics, Imperial College London, London, UK; 3 Institute of Genetic Medicine, Central Parkway, Newcastle University, Newcastle Upon Tyne, UK; 4 Nuffield Department of Clinical Neurosciences, John Radcliffe Hospital, University of Oxford, Oxford, UK; 5 National CJD Research & Surveillance Unit, University of Edinburgh, Western General Hospital, Edinburgh, UK; 6 Department of Basic and Clinical Neuroscience, Institute of Psychiatry, Psychology and Neuroscience, King’s College London, De Crespigny Park, London, UK; 7 Institute of Neuroscience, Newcastle University, Campus for Aging and Vitality, Newcastle upon Tyne, UK; 8 MRC Mitochondrial Biology Unit, University of Cambridge, Cambridge, UK

**Keywords:** somatic variant, brain, neurodegenerative disorders, exome sequencing, embryogenesis

## Abstract

**Purpose:**

To systematically study somatic variants arising during development in the human brain across a spectrum of neurodegenerative disorders.

**Methods:**

In this study we developed a pipeline to identify somatic variants from exome sequencing data in 1461 diseased and control human brains. Eighty-eight percent of the DNA samples were extracted from the cerebellum. Identified somatic variants were validated by targeted amplicon sequencing and/or PyroMark® Q24.

**Results:**

We observed somatic coding variants present in >10% of sampled cells in at least 1% of brains. The mutational signature of the detected variants showed a predominance of C>T variants most consistent with arising from DNA mismatch repair, occurred frequently in genes that are highly expressed within the central nervous system, and with a minimum somatic mutation rate of 4.25 × 10^−10^ per base pair per individual.

**Conclusion:**

These findings provide proof-of-principle that deleterious somatic variants can affect sizeable brain regions in at least 1% of the population, and thus have the potential to contribute to the pathogenesis of common neurodegenerative diseases.

## Introduction

Pathogenic genetic variants affecting over 50 nuclear genes contribute to the pathogenesis of late onset neurological disorders.^1^ Present in every cell in the body, these genetic variants are either inherited or arise through a de novo variant in the gamete. In contrast, some age-related disorders such as cancer arise through the accumulation of somatic variants within a cell lineage during life, creating genetic heterogeneity within a tissue or organ (somatic mosaicism). Almost half of these variants arise decades before tumor initiation,^2–4^ raising the possibility that somatic variants acquired by a similar process during development are also present within nonmalignant human tissues. Within the nervous system, somatic variants have been identified in rare, early onset, focal neurological disorders such as hemimegalencephaly and lissencephaly,^5–8^ demonstrating that protein-coding variants with mosaic allelic fractions as low as 8% in the brain can cause macroscopically overt structural neurological diseases,^6^ though even lower allelic fractions of around 1% may cause milder phenotypes such as focal cortical dysplasia.^9^ To date, however, the frequency of somatic variants in the human brain, and particularly in those late onset neurological disorders, has not been studied systematically.

## Material and methods

### Brain samples

**Fig. 1:**
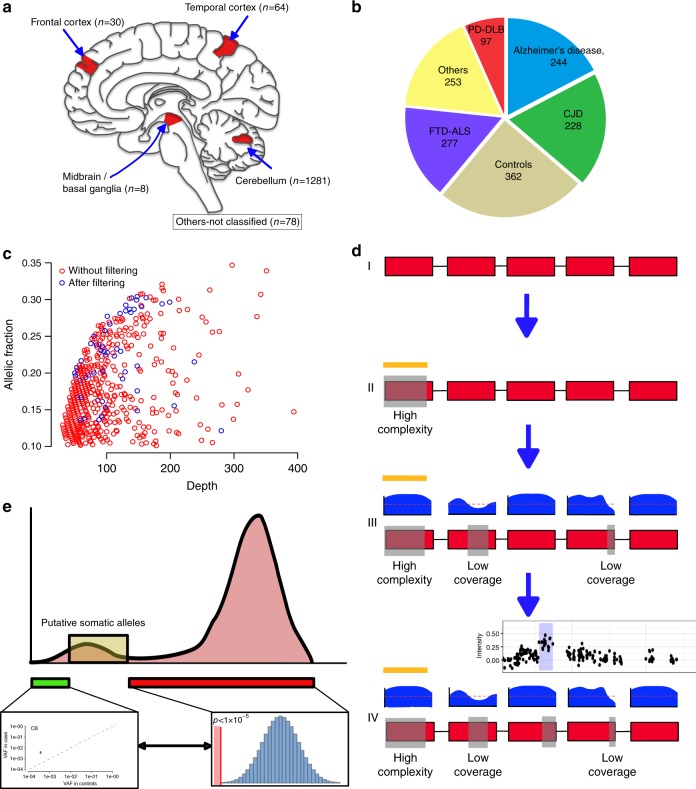
**Detection of somatic variants in 1461 postmortem human brains.** (**a**) Brain regions sampled within the study. (**b**) The proportion and number of individuals in each cohort. (**c**) Unfiltered variant allele fraction (VAF) with between 10% and 35% against relative exome sequencing depth. Those that were present before and after filtering are shown (red and blue respectively). (**d**) Variant detection pipeline. Section I: Exons are shown in red, with intergenic and intronic regions as a black line. II: Regions of high genomic complexity and common structural variants (determined from population databases and previous studies) were removed (yellow line/gray box). III: Relative sequencing depth of each exon is shown in blue above the relevant exon. Bases in which the sequencing depth was below 30 (as depicted by the red dashed line) in an individual were removed. These regions are then shown by gray boxes on the schematic exome and were also removed. IV: Finally, regions in which copy-number variants (gains or losses) were called from array genotyping^10^ were also removed from the overall panel. An example plot of the array genotyping in which a copy-number gain has been detected is shown. Again the corresponding region was removed from the exome depicted by a gray box on the exome panel. After these steps, remaining regions were subsequently subjected to analysis by deepSNV and a binomial test against the mean VAF for heterozygous variants (47%). (**e**) Schematic representation of the putative somatic alleles in the data set. A distribution of VAF in the whole data set is shown (pink histogram). Putative somatic alleles were those in which the VAF was greater than base error rate (as determined from deepSNV [green box and linked inset]), and those that also differed from the binomial threshold (<1 × 10^−5^) compared with an assumed VAF of 47% for heterozygosity. *CJD* Creutzfeldt–Jakob disease, *PD-DLB* Parkinson disease and dementia with Lewy bodies

Ethical approval for the genetic analysis of postmortem brain tissue was obtained from the ethical review board of each participatingcenter. DNA was extracted from 1461 human brains (cerebellum: *n* = 1281 [87.7%], cerebral cortex: *n* = 94 [6.5%], basal ganglia: *n* = 8 [0.5%], not classified: *n* = 78 [5.3%]) from 1099 patients with neurodegenerative diseases including Alzheimer disease, frontotemporal dementia or amyotrophic lateral sclerosis (FTD-ALS), Creutzfeldt–Jakob disease (CJD), Parkinson disease and dementia with Lewy bodies (PD-DLB), and 362 age-matched controls within the Medical Research Council (MRC) UK Brain Bank Network. Controls were defined as having no antemortem history of neurological disease, no neuropathological features of any neurodegenerative disease, and a Braak neurofibrillary tangle stage of ≤2 (Fig. 1a, b, Supplementary Material Table 1 for demographics and clinical data). The characteristics of the study group have been described previously.^10^ Brain regions were sampled from available brain regions with the maximum DNA extraction yield per milligram of tissue.

### Exome sequencing (ES) and somatic variant calling

Exome sequencing was performed on all samples as previously described.^10^ Sequencing data was aligned against the University of California–Santa Cruz (UCSC) hg19 human reference genome using Burrows–Wheeler Aligner (BWA).^11^ GATK’s Haplotype Caller from Genome Analysis Toolkit (GATK version 3.4) was used to determine allelic counts and genotypes across the genome.^12^ We excluded the following regions within quality control: (1) regions with the higher likelihood of misalignment and polymerase chain reaction (PCR) artifacts in the genome;^2^ (2) specific small copy-number variants (CNVs) in 1321 individuals called by array genotyping;^10^ and (3) sites with read depth <30× in any sample (Fig. 1c, d, Supplementary Material Figs. 1 and 2). This resulted in a total of 5,906,849 base pairs (bp) per individual available for subsequent analysis.

To detect putative somatic variants, we used a modified workflow that was initially described by Genovese et al.,^2^ but this time using a pan-exome approach. Firstly, we restricted variants to single-nucleotide variants (SNVs) and excluded all variants with the relatively high variant allele fraction (VAF, the ratio of variant allele: total allele) >50% or <10% (Fig. 1c). VAFs were subsequently identified that significantly differed from the mean VAF for heterozygous variants (47% in our data set, binomial test *P* *<* 1 × 10^−5^) (Fig. 1e). We also excluded those variants present more than once in the cohort, and those with a minor allelic frequency (MAF) >0.5% within the ExAC database of Human Exome Variation^13^ (Supplementary Material Fig. 3).

To confirm that detected putative somatic alleles also significantly differed from the base error rate in addition to the mean allelic frequency for a heterozygous variant, we utilized deepSNV^14,15^ to compare the nucleotide counts for each putative somatic variant against 328 random samples within the same data set. Relative read counts were retrieved from the BAM file of each case, and the individual of interest was compared against the variant allele counts for the other 328 individuals using a β-binomial distribution. Variants with a *p* value <0.001 were included as putative somatic variants. This ensured putative somatic alleles passing both thresholds differed from both the observed VAF of heterozygous variants, and from the local base error rate (Fig. 1e). All putative somatic variants were confirmed by inspection in Integrative Genomic Viewer^16,17^ and were annotated using ANNOVAR^18^ (Supplementary Material Fig. 2).

### Variant validation

Variants remaining after the above filtering strategy were then validated by targeted amplicon sequencing to confirm a somatic variant in cases, together with their absence from controls (VAF<1%). Specific primers spanning putative somatic alleles were designed using NCBIPrimerBLAST (https://www.ncbi.nlm.nih.gov/tools/primer-blast/). Amplicons were generated that spanned the putative somatic variant, and were sequenced in the sample containing the putative somatic allele and in a control case with DNA extracted from the same brain region. PCRs were performed using MyTaq HS polymerase (Bioline, USA), and pooled amplicons were sequenced using MiSeq Reagent Kit v3.0 (Illumina, CA, USA) with paired-end, 150-bp reads. FASTQ files were analyzed using in-house bioinformatic pipelines. Reads were aligned to the UCSC hg19 human genome reference using BWA.^11^ Variant calling was performed using GATK’s Haplotype Caller^12^ (minimum depth = 500×, minimum supporting reads = 40, base quality ≥30 and mapping quality ≥20), and variant to reference allelic frequencies manually extracted from BAM files. Subsequently, all validated variants were manually inspected and confirmed in Integrative Genomic Viewer (IGV)^16,17^ (Supplementary Material Fig. 2).

Five variants from five cases fulfilling the above criteria were also randomly selected for validation by PyroMark^®^ Q24 using standard protocols (Qiagen Inc). Data was analyzed using the PyroMark Q24 software for AQ quantitation, with relevant allelic frequencies determined from the sequencing pyrogram. Each sample and control was run in duplicate and the mean of the VAF determined for each allele in each sample and control.

### Occurrence of somatic variants at methylated bases

We downloaded genome bisulfite sequencing (GBS) data from the inner cell mass (ICM) of an early developmental human embryo.^19^ In total, 476,286,624 of 3,095,693,981 total bases were methylated (15.4%). We subsequently sought to determine whether there was enrichment of somatic mutagenesis at methylated sites by performing a binomial test using 15.4% as the background probability against the proportion of validated variants that occurred at methylated bases.

### Mutational spectra and signatures

Mutational spectra were derived directly from the reference and alternative allele at each somatic variant allele. To understand the potential mechanisms of somatic mutagenesis we compared the somatic mutation spectrum and triplet allele (reference allele either side of the somatic allele) against 30 previously defined mutational signatures in cancer^20^ and against the mutational signatures to de novo genetic variants derived from trio studies in the population.^21^

### Variants in the brain proteome

All gene expression data was downloaded from the Human Protein Atlas,^22^ and each gene containing a somatic variant was annotated according to the expression classification within the brain. Genes were classed as either (1) Elevated in brain, (2) Expressed in all, (3) Mixed expression pattern, (4) Not detected in brain, or (5) Not detected in any tissue as determined by the Human Protein Atlas. Binomial testing was performed in R (v3.3) (http://CRAN.R-project.org/) to determine whether genes containing somatic variants were significantly different from the expression profile of all genes across the human genome within these five categories.

### Conserved genes

To determine the relative constraint for missense variation within the germline for each gene containing a somatic variant, we annotated each gene with the missense *z*-score as determined by the Exome Aggregation Consortium (ExAC).^13^ Binomial testing was performed to compare the proportion of genes within each quartile of the spectrum of missense constraint as determined by ExAC in R.

### Data availability

Clinical, pathological, and genetic data from this study have been submitted to the European Genome-phenome Archive (EGA, https://www.ebi.ac.uk/ega/home) under accession number EGAS00001001599 (password available on request). VCF files and associated and annotated metadata (clinical and neuropathological diagnosis, age of disease onset, and age of death) are available for download through this archive. All requests for data should be made to the Data Access Committee as identified through http://www.mrc.ac.uk/research/facilities/, http://www.mrc.ac.uk/research/facilities/brain-banks/.

## Results

### Characteristics of variants

**Fig. 2:**
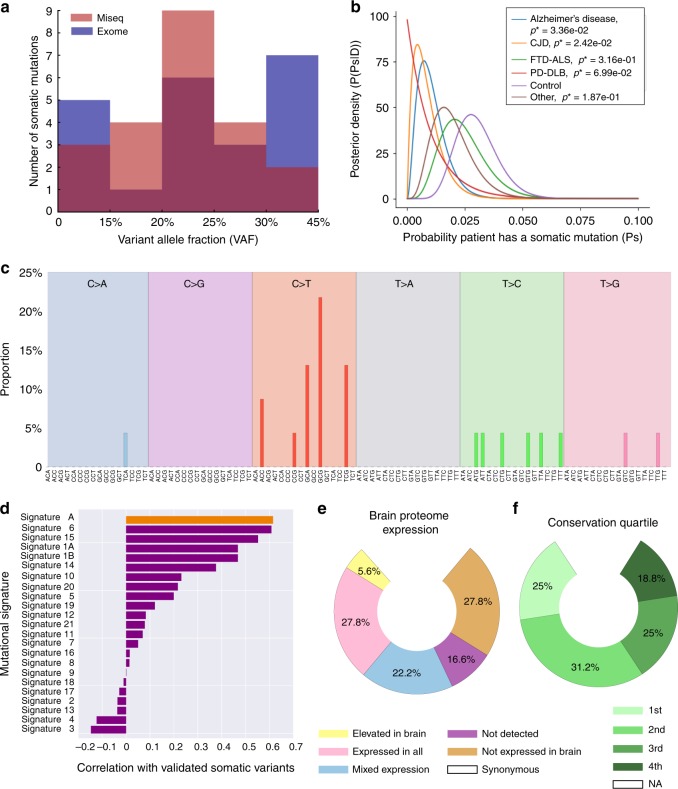
**Distribution and mutational profile of the validated somatic variants.** (**a**) Distribution of allele frequencies for the validated variants in the study are shown, with the relative variant allele frequency (VAF) for each allele as detected on both the MiSeq (pink), exome sequencing (blue), and overlapping between two platforms (purple) shown. (**b**) Probability of a variant occurring in each cohort assuming a uniform prior probability and that each person is a Bernoulli trial with probability *p* of developing a pathogenic variant. (**c**) Mutational signature of all validated somatic variants. The mutated allele plus the flanking 3′ and 5′ base are shown. (**d**) Correlation between the mutational signature of validated somatic variants and the mutational profiles observed in de novo germline variants detected in the population^21^ (top orange bar, signature A) and 21 forms of cancer^20^ (purple bars). The probable disease associations, or type of cancer in which the signature was detected by Alexandrov^20^ are shown next to the signature number. The Pearson correlation coefficient is shown for each signature. (**e**) Proportion of validated variants within genes grouped by brain proteome expression.^22^ (**f**) Proportion of validated variants based on each quartile of the gene conservation scores within the germline (4th quartile being the most conserved in the germline). *CJD* Creutzfeldt–Jakob disease, *FTD-ALS* frontotemporal dementia or amyotrophic lateral sclerosis, *PD-DLB* Parkinson disease and dementia with Lewy bodies

Exome sequencing was performed on 1461 human brain samples from 1099 patients with neurodegenerative diseases and 362 age-matched controls (Fig. 1a, b, Supplementary Material Table 1). Mean sequencing depth of ES from 1461 samples was 51.9-fold (SD = 12.9), with no significant difference between any disease or controls (one-way analysis of variance [ANOVA] test *p* >0.05) (Supplementary Material Fig. 1). Using the described filtration steps we detected 56 somatic variants in 46 brains (3.2% of 1461) (Supplementary Material Table 2). Specific short primer sequences were able to be designed for 40 of the 56 variants using two orthogonal methods (Supplementary Material Fig. 2), and confirmed the presence of a somatic variant in 22 (55.0%) of the tested alleles; a confirmation rate in keeping with other studies of somatic variation^23^ (Fig. 2a, Table 1, Supplementary Material Fig. 4). The majority of validated variants were transitions (86.4%, *n* = 19) with 23.4% (*n* = 3) transversions. C>T variants were by far the most common (59.1%) (ref. ^24^), and 27.2% (*n* = 6/22) of the validated variants occurred at bases methylated in the inner cell mass.^19^ In addition, 8 of the 13 C>T pathogenic variants (61.5%) were present at CpG sites within the genome. None of the identified somatic variants were seen in the heterozygote state in the 1461 brains, and all were extremely rare in the background population.^13^ There was also no difference in the frequency of somatic variants between the different disease and control groups (Fisher exact test *p* > 0.05) (Fig. 2b) indicating that, whilst mutational rates may not be increased in patients with neurodegenerative diseases compared with healthy aged individuals, somatic variants at high variant allele frequencies are relatively common in the human brain.

**Table 1:** Validated somatic variants in 1461 human brains

Variant data														Clinical data			
Chromosome	Base position	Ref allele	Alt allele	Mutation	Gene	AA change	ExAC	SIFT score	SIFT	Human Proteome expression	Conservation quartile	Methylated in ICM	Exome VAF	Sample ID	Gender	Brain region	Disease group
chr1	248224344	C	T	Non-syn	*OR2L3*	p.R121C	4.94E-05	0.02	D	Not detected	2	N	15.2%	1	M	Cerebellum	Control
chr7	150815676	C	T	Non-syn	*AGAP3*	p.S81L	N/A	0.02	D	Expressed in all	4	N	22.3%	2	M	Temporal cortex	Control
chr11	104905100	T	G	Non-syn	*CASP1*	p.K37Q	4.10E-03	0.59	T	Expressed in all	2	N	16.9%	3	M	Cerebellum	Control
chr17	39502849	T	G	Non-syn	*KRT33A*	p.R316S	1.10E-03	0.67	T	Not expressed brain	2	N	22.1%	4	M	Cerebellum	CJD
chr3	45837911	T	C	Start lost	*SLC6A20*	p.M1V	8.77E-05	0.43	T	Not expressed brain	3	N	20.3%	5	M	Cerebellum	Alzheimer disease
chr3	122629742	T	C	Non-syn	*SEMA5B*	p.H59R	N/A	0.02	D	Elevated brain	4	N	19.3%	5	M	Cerebellum	Alzheimer disease
chr19	36275201	G	A	Non-syn	*ARHGAP33*	p.A517T	N/A	0.13	T	Mixed expression	3	N	26.0%	6	M	Cerebellum	Other (PSP)
chr12	6138596	C	T	Non-syn	*VWF*	p.R960P	8.24E-06	0.26	T	Mixed expression	3	N	19.5%	7	F	Cerebellum	Control
chr11	56344581	G	T	Non-syn	*OR5M10*	p.T206N	1.20E-03	1	T	Not detected	2	N	13.0%	8	F	Cerebellum	Other (epilepsy)
chr16	4833750	A	G	Non-syn	*SETP12*	p.I131T	1.68E-05	0.01	D	Not expressed brain	1	N	22.8%	9	M	Frontal cortex	Control
chr7	1535876	C	T	Non-syn	*INTS1*	p.D671N	8.26E-06	0	D	Expressed in all	3	N	14.7%	9	M	Frontal cortex	Control
chr8	144921555	T	C	Non-syn	*NRBP2*	p.I171V	3.32E-04	0.15	T	Expressed in all	2	N	30.4%	9	M	Frontal cortex	Control
chr2	85991195	C	T	Non-syn	*ATOH8*	p.R284W	8.30E-06	0	D	Mixed expression	4	Y	28.3%	10	F	Temporal cortex	Control
chr1	24125194	G	A	Non-syn	*GALE*	p.R50W	1.66E-05	0.02	D	Expressed in all	2	Y	21.7%	11	F	Cerebellum	Control
chr1	17570577	T	C	Non-syn	*PADI1*	p.C126R	N/A	0.01	D	Not expressed brain	1	N	25.3%	12	M	Cerebellum	Other (dementia)
chr17	76499013	G	A	Syn	*DNAH17*	N/A	4.05E-03	N/A	N/A	Not expressed brain	N/A	Y	28.2%	12	M	Cerebellum	Other (dementia)
chr11	1718844	T	C	Syn	*KRTAP5–6*	N/A	1.65E-05	N/A	N/A	Not detected	N/A	N	14.9%	13	F	Cerebellum	FTD-ALS
chr19	9361855	G	A	Non-syn	*OR7E24*	p.A46T	2.50E-05	0	D	Not expressed brain	1	Y	30.2%	13	F	Cerebellum	FTD-ALS
chr20	60888258	G	A	Syn	*LAMA5*	N/A	N/A	N/A	N/A	Expressed in all	N/A	N	23.0%	13	F	Cerebellum	FTD-ALS
chr16	88712548	G	A	Syn	*CYBA*	N/A	N/A	N/A	N/A	Expressed in all	2	Y	23.5%	14	F	Cerebellum	Control
chr22	50752254	G	A	Non-syn	*DENND6B*	p.R398W	1.66E-05	0	D	Mixed expression	1	Y	20.2%	15	M	Cerebellum	FTD-ALS
chr6	5004177	G	A	Syn	*RPP40*	N/A	4.12E-05	N/A	N/A	Expressed in all	N/A	N	22.9%	15	M	Cerebellum	FTD-ALS

Variant data shows the chromosome, base position, and reference and alternate allele (hg19 build), together with the amino acid change, frequency in the ExAC population data set,^13^ SIFT annotation score and classification,^24^ expression cohort in the Human Proteome Atlas,^21^ the quartile of genetic conservation within the human genome,^13^ presence of methylation at that base in the ICM of an early developmental human embryo,^18^ and the VAF in the exome sequencing (ES) data. Clinical data for each individual comprising sample ID, gender, brain region, and disease group are shown.

*AA* amino acid, *CJD* Creutzfeldt–Jakob disease, *D* deleterious, *FTD-ALS* frontotemporal dementia–amyotrophic lateral sclerosis, *ICM* inner cell mass, *PD-DLB* Parkinson disease–dementia with Lewy bodies, *N/A* not applicable, *Non-syn* nonsynonymous, *PSP* progressive supranuclear palsy, *Syn* synonymous, *T* tolerated, *VAF* variant allele frequency

### Mutational spectrum and signatures

We further examined the correlation between the observed signature of base mutagenesis with the signature observed in cancer,^20^ observing the strongest correlation with variants thought to be due to mismatch repair errors occurring during DNA replication and recombination (Pearson product moment test *r*^2^ = 0.61, *p* = 5.02 × 10^−11^) (Fig. 2c, d). The data were also compared with mutational profile of de novo germline variants in the population derived from the de novo db mutation database,^21^ also revealing a strong association with the mutational profile of de novo variation (Pearson product moment test *r*^2^ = 0.62, *p* *=* 2.74 × 10^−11^) (Fig. 2c, d).

### Pattern of gene expression and selection pressure

We subsequently determined the tissue expression pattern of each gene in which a somatic variant was observed, and saw that ten (58.8%) of the nonsynonymous or start-loss variants were present in genes expressed within the brain. These data are consistent with the notion that the somatic variants were not selected against based on tissue expression, and were equally distributed across the expression profile of the human genome. This raises the possibility that somatic variants contribute to disease pathogenesis in several human tissues, including the brain (Fig. 2e, Supplementary Material Table 3). Although speculative, VAF of the observed somatic variants could actually reflect positive selection of some variants, particularly if they arose in later stages of development.

We also found no evidence that the selection pressures seen within the germline also act on the somatic variants we observed in the brain, with nonsynonymous somatic variants evenly distributed across conserved and nonconserved regions of the human genome (binomial test *p* = NS) (Fig. 2f).

Finally, we determined that 58.8% of the nonsynonymous or start-loss variants (10/17) were predicted to be deleterious by SIFT^25^ suggesting that they are highly likely to have detrimental effects on gene expression (Table 1). When taken together, these findings suggest that somatic variants in the brain may not been subject to the same constraints as genetic variation in the germline,^26^ rendering all regions of the brain exome vulnerable to somatic mutagenesis, and therefore potentially conferring the possibility of causing a wide range of neurodegenerative diseases.

### Estimates of the mutation rate in human brains

**Fig. 3:**
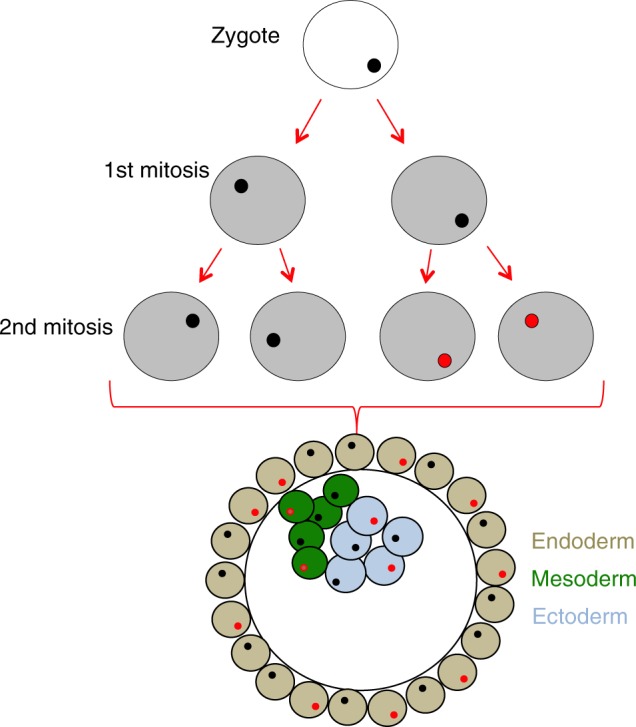
**Early cell division after fertilization.** Schematic diagram showing early embryonic development. An example of somatic variant (red) is shown, with the subsequent distribution of this variant within the embryo

To determine the somatic mutation rate observed within the human brain we first assumed that the variants occurring within the first two cell divisions of the human zygote would give rise to VAF of 10–30%, and would likely be present in all human tissues, having arisen before tissue differentiation^27^ (Fig. 3). In this study, after quality control (QC) and the removal of structural variation, we analyzed 5,906,849 nucleotide bases in each individual brain (see Methods). Across the whole cohort (*n* = 1461 cases), this resulted in the analysis of 8,629,906,389 nucleotide bases, which contained 22 validated somatic variants. This equates to a mutation rate of 2.55 × 10^−9^. Assuming that the detectable variants occur at either the first or second cell divisions (corresponding to an allelic fraction of 0.25 and 0.125 respectively, and arising from a total of six cells; Fig. 2a, Fig. 3), this results in a minimum somatic mutational rate across the human exome of 4.25 × 10^−10^ per base pair per individual in the first two cell divisions of the human zygote. This is slightly lower than previously calculated human somatic mutation rates of 2.67 × 10^−9^ (ref. ^26^), endorsing the sensitivity of our approach. Finally, assuming 3 billion bases in the full human genome, our data suggest that ~1.3 somatic variants across the whole genome will occur during the first two cell divisions (3 × 10^9^ multiplied by 4.25 × 10^−10^). This is slightly lower than recent estimates using genome sequencing where ~3 variants were estimated to occur per cell per division in very early development.^23^ This difference could reflect methodological differences such as the particularly conservative nature of our validation algorithm, or be due to a lower mutation rate across the human exome when compared with noncoding regions.

## Discussion

These data are the first to quantify the degree of high-level (VAF >10%) somatic mosaicism within the human brain, and show that at least 1% of people possess a somatic protein-coding variant within the central nervous system. Given the close correlation between our observed somatic mutation rate and previous estimates, when extrapolated across the whole genome (of 3 billion bases), our data suggest that each human brain may possess at least ~1.3 high-frequency (>10% VAF) somatic variants that have arisen during the first two embryonic cell divisions. When considered alongside the slightly higher mutation rates within the male germline of 1.28 × 10^−8^, which confers an average of 76.9 de novo germline variants in each individual,^28^ then the degree of nonanticipated inherited or acquired genetic variation within an individual can be extensive (~80 alleles). This has important implications in considering the potential genetic etiology of human neurological diseases.

Whilst the number of validated somatic protein-coding variants in our study was small at 22, we saw no evidence of the same selective constraints seen within the germline, which would otherwise limit the number of potentially detrimental germline alleles acquired during development.^13^ Given the predominance of C>T somatic variants, the observation that 27.2% (*n* = 6/22) of the validated variants occurred at bases methylated in the inner cell mass (Table 1) (ref. ^19^) implicates the deamination of methylated cytosines as one potential mechanism, particularly given the enrichment for C>T variants at CpG sites. It was also surprising that there was a relatively strong association with the mutational signatures seen with de novo mutagenesis within the germline,^21^ suggesting that similar mechanisms of mutagenesis may be involved in the formation of these variants,^23^ albeit that they do not appear to be selected against in the brain.

A second possibility is that the detected variants were truly focal within the human brain, having arisen during corticogenesis, and subsequent to tissue differentiation during embryogenesis. For example, Poduri et al.^7^ detected a focal somatic variant with a VAF of 17% within the brain causing hemimegalencephaly that was not present in the patient’s blood. Without additional tissue samples from other organs we cannot exclude this possibility in the cases we studied here. However, the lack of bias for detectable mosaicism in any of the brain region samples (cerebellum; 17/22 (Fisher exact test versus other brain regions *p* *=* 0.18) (Fig. 1a, Table 1), together with the lack of focal morphological abnormalities such as those observed by Poduri et al., point toward an early developmental origin rather than a late focal origin for the variants we report here. However, we do appreciate that we cannot confirm this directly. These problems are likely to be overcome by large scale, higher depth sequencing that will detect lower levels of mosaicism. This will refine the mutation rates and clarify the origin of variants within individuals with neurodegenerative disorders. However, based on the data we report here, mosaicism should also be considered as a potential source of unexpected genetic findings following diagnostic exome and genome sequencing in neurological disorders.

It should be noted that 88% of the DNA samples studied were extracted from the cerebellum, with no enrichment for cerebellar or noncerebellar extraction sites within any disease group or controls. It will be important to validate these findings in other brain regions. This is particularly relevant for the investigation of neurodegenerative diseases where there is little in the way of cerebellar pathology. Nonetheless, we have demonstrated that at least 1% of human brain samples contain high-level somatic variants present in at least 10% of cells. Many of these variants were extremely rare in the germline of the population, were highly expressed within the brain, and conferred the ability to markedly alter protein function. Based on the observed mutational signatures, we determine that they are likely to be driven by DNA mismatch repair, and assuming an early developmental origin, are consistent with a somatic mutation rate in the human exome of at least 4.25 × 10^−10^ per base pair per individual. Taken together these data determine the frequency, nature, and likely origin of high-frequency somatic variants in the human brain and show how they have the potential to contribute to a range of neurological disorders.

## Electronic supplementary material

Supplementary Data
