# Supplementary material for: Frequency and signature of somatic variants in 1461 human brain exomes
Source: Genet Med. 2018 Sep 14;21(4):904–12. doi: 10.1038/s41436-018-0274-3 (PMC6544539; doi:10.1038/s41436-018-0274-3)
Supplement: Supplementary file 1 — Supplementary Data [file 41436_2018_274_MOESM1_ESM.pdf]

Supplementary Data

Supplementary Table 1. Clinical and demographic details of all samples within the study.

Disease Group	Total cohort	Male (n) (%)	Female (n) (%)	Age onset (years) (SD)	Age Death (years) (SD)	Disease duration (years)
**Alzheimer's disease**	277	131 (47.3)	146 (52.7)	65.4 (10.2)	77.7 (11.7)	7.3 (4.7)
**CJD**	228	123 (53.9)	105 (46.1)	52.0 (20.1)	53.0 (19.6)	1.0 (1.0)
**Control**	362	232 (64.1)	130 (35.9)	N/A	63.3 (18.9)	N/A
**FTD-ALS**	244	143 (58.6)	101 (41.4)	59.4 (11.8)	64.6 (11.7)	6.5 (5.6)
**Other**	253	131 (51.8)	122 (48.2)	66.7 (20.2)	79.7 (14.3)	10.6 (15.8)
**PD-DLB**	97	64 (66.0)	33 (34.0)	63.7 (10.1)	74.9 (8.2)	9.2 (5.8)
**Total**	1461	824	637	59.3 (17.8)	68.2 (18.0)	5.6 (8.9)

Abbreviations: CJD, Creutzfeldt Jakob Disease; FTD-ALS, Frontotemporal dementia – Amyotrophic lateral sclerosis; PD-DLB, Parkinson's disease – Dementia with Lewy Bodies.

Supplementary Table 2. Detected putative somatic variants from exome sequencing

No.	Chr	Pos	Ref	Alt	VAF	Gene name	Nonsynonymous/ synonymous SNV	Validation outcome
1	chr1	17570577	T	C	25.29%	PADI1	nonsynonymous SNV	Validated
2	chr1	24125194	G	A	21.67%	GALE	nonsynonymous SNV	Validated
3	chr1	43166558	C	T	17.57%	YBX1	nonsynonymous SNV	Technical failure
4	chr1	46872017	C	T	19.67%	FAAH	nonsynonymous SNV	Heterozygous
5	chr1	108742671	T	G	27.19%	SLC25A24	synonymous SNV	Heterozygous
6	chr1	228447313	C	G	28.26%	OBSCN	nonsynonymous SNV	Technical failure
7	chr1	248224344	C	T	15.15%	OR2L3	nonsynonymous SNV	Validated
8	chr10	96535189	G	C	27.72%	CYP2C19	nonsynonymous SNV	Heterozygous
9	chr11	1258327	G	A	25.41%	MUC5B	nonsynonymous SNV	Not present
10	chr11	1718844	T	C	14.86%	KRTAP5-6	synonymous SNV	Validated
11	chr11	56344581	G	T	13.04%	OR5M10	nonsynonymous SNV	Validated
12	chr11	104905100	T	G	16.90%	CASP1	nonsynonymous SNV	Validated
13	chr12	6138596	C	T	19.54%	VWF	nonsynonymous SNV	Validated
14	chr12	9243951	A	G	14.71%	A2M	nonsynonymous SNV	Not present
15	chr12	42512876	T	G	16.13%	GXYLT1	nonsynonymous SNV	Technical failure
16	chr12	104376635	C	T	18.52%	TDG	synonymous SNV	Not present
17	chr13	21742240	C	A	22.95%	SKA3	nonsynonymous SNV	Heterozygous
18	chr14	23844983	G	A	27.34%	IL25	nonsynonymous SNV	Heterozygous
19	chr16	4833750	A	G	22.77%	SETP12	nonsynonymous SNV	Validated
20	chr16	88712548	G	A	23.53%	CYBA	synonymous SNV	Validated
21	chr17	13400048	G	C	27.40%	HS3ST3A1	nonsynonymous SNV	Heterozygous

22	chr17	39502849	T	G	22.06%	KRT33A	nonsynonymous SNV	Validated
23	chr17	39521517	C	T	29.93%	KRT33B	synonymous SNV	Heterozygous
24	chr17	45234387	A	G	16.85%	CDC27	nonsynonymous SNV	Not present
25	chr17	48070896	C	A	30.25%	DLX3	nonsynonymous SNV	Technical failure
26	chr17	76499013	G	A	28.24%	DNAH17	synonymous SNV	Validated
27	chr19	8999476	G	C	20.21%	MUC16	nonsynonymous SNV	Heterozygous
28	chr19	9006365	C	A	17.24%	MUC16	nonsynonymous SNV	Not present
29	chr19	9361855	G	A	30.15%	OR7E24	nonsynonymous SNV	Validated
30	chr19	14877162	T	C	20.00%	EMR2	synonymous SNV	Technical failure
31	chr19	36275201	G	A	26.04%	ARHGAP33	nonsynonymous SNV	Validated
32	chr19	50170347	G	A	22.34%	BCL2L12	nonsynonymous SNV	Technical failure
33	chr2	26702178	C	T	28.11%	OTOF	nonsynonymous SNV	Heterozygous
34	chr2	85991195	C	T	28.30%	ATOH8	nonsynonymous SNV	Validated
35	chr20	60718900	C	T	19.70%	SS18L1	synonymous SNV	Technical failure
36	chr20	60888258	G	A	22.95%	LAMA5	synonymous SNV	Validated
37	chr21	44836731	C	T	27.42%	SIK1	nonsynonymous SNV	Technical failure
38	chr22	50752254	G	A	20.16%	DENND6B	nonsynonymous SNV	Validated
39	chr3	45837911	T	C	20.31%	SLC6A20	start lost	Validated
40	chr3	122629742	T	C	19.28%	SEMA5B	nonsynonymous SNV	Validated
41	chr5	115177753	A	G	18.18%	AP3S1	nonsynonymous SNV	Technical failure
42	chr5	140242885	C	G	27.56%	AX746964	nonsynonymous SNV	Heterozygous
43	chr6	5004177	G	A	22.92%	RPP40	synonymous SNV	Validated
44	chr6	26458871	T	C	22.45%	BTN2A1	nonsynonymous SNV	Technical failure
45	chr6	54066945	A	C	18.18%	MLIP	nonsynonymous SNV	Heterozygous

46	chr6	56497767	A	C	14.81%	DST	nonsynonymous SNV	Technical failure
47	chr6	136594277	G	A	20.83%	BCLAF1	nonsynonymous SNV	Technical failure
48	chr7	1535876	C	T	14.74%	INTS1	nonsynonymous SNV	Validated
49	chr7	2617927	C	A	15.35%	IQCE	nonsynonymous SNV	Technical failure
50	chr7	150815676	C	T	22.32%	AGAP3	nonsynonymous SNV	Validated
51	chr7	157178323	C	T	26.15%	DNAJB6	nonsynonymous SNV	Technical failure
52	chr8	144921555	T	C	30.41%	NRBP2	nonsynonymous SNV	Validated
53	chr8	144940260	C	T	12.14%	EPPK1	nonsynonymous SNV	Technical failure
54	chr9	95481489	C	T	26.92%	BICD2	nonsynonymous SNV	Heterozygous
55	chrX	135960166	G	A	25.25%	RBMX	nonsynonymous SNV	Heterozygous
56	chrX	153416209	C	T	21.17%	OPN1LW	nonsynonymous SNV	Technical failure

The 56 putative somatic variants detected in this study. Chromosome, base position (with reference to hg19 build), reference and observed alternate allele, ratio of alternate to reference allele, gene name, and whether the variant is synonymous or non-synonymous are shown. Finally, the results of the validation experiment are shown. Abbreviations: Chr, Chromosome; Pos, Position; Ref, Reference allele; Alt, Alternative allele; VAF, Variant Allele Frequency.

Supplementary Table 3. Expected and observed variants occurring within different cohorts of genes as grouped by established brain expression data.

Brain proteome data			
**Brain proteome gene set**	**Observed variant (total validated)**	**Expected variant frequency**	**binomial test (p-value) ***
**Elevated in Brain (n=1224)**	1	1.1	1.0
**Expressed in all (n=8588)**	5	7.8	0.48
**Mixed expression pattern (n=4404)**	4	4	1.0
**Not detected in brain (n=1318)**	2	1.2	0.36
**Not detected in any tissue (n=4157)**	5	3.8	0.56

*Binomial testing was performed between observed and expected variant proportions.

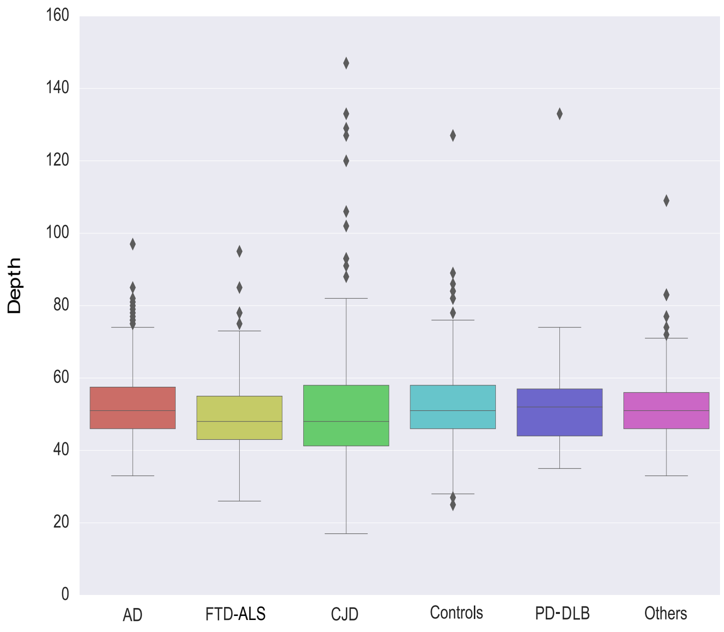

Supplementary Figure 1. Average sequencing depth for each group within the study. There were no significant differences (one-way ANOVA). Abbreviations: AD, Alzheimer's disease; CJD, Creutzfeldt Jakob Disease; FTD-ALS, Frontotemporal dementia – Amyotrophic lateral sclerosis; PD-DLB, Parkinson's disease – Dementia with Lewy Bodies.

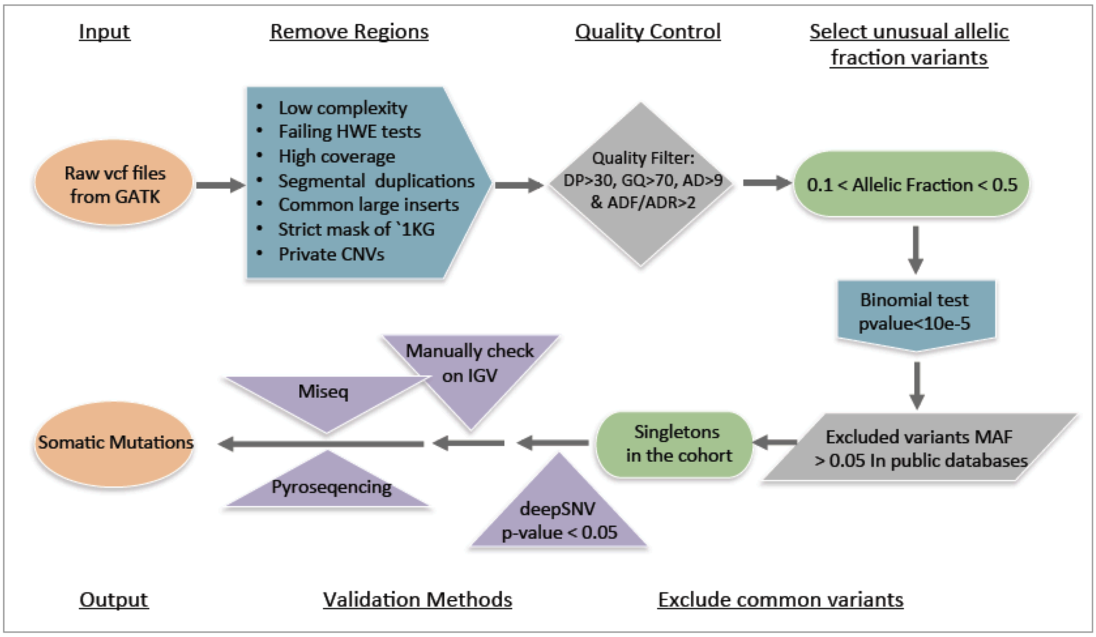

Supplementary Figure 2. An overview of the pipeline to detect somatic variants within the dataset. Abbreviations: VCF, Variant Call Format; GATK, Genome Analysis Tool Kit; HEW, Hardy-Weinberg; CNV, Copy Number Variant; DP, Depth; GQ, Genotype Quality; AD, Allele Depth; ADF/ADR, Depth of bases supporting variant on forward/reverse strand; MAF, Minor Allele Frequency.

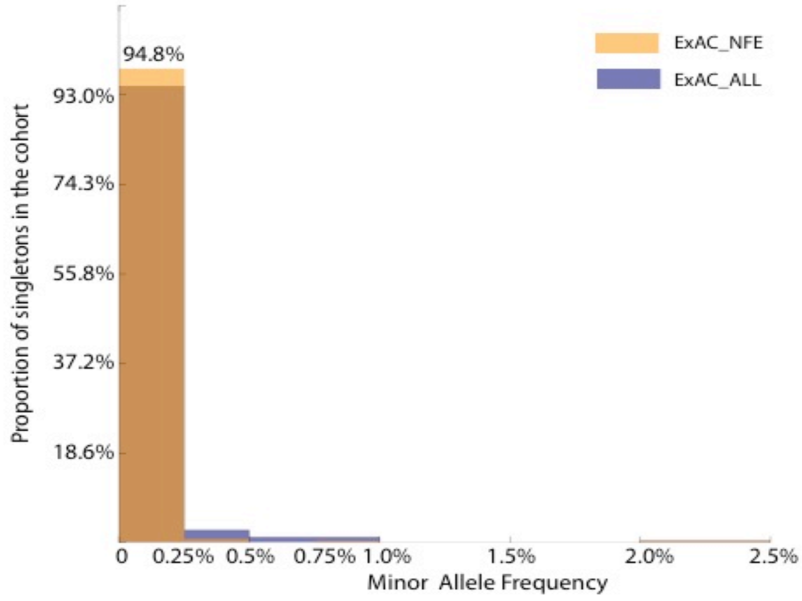

Supplementary Figure 3. Proportion of putative somatic variants (before further filtering) against their minor allele frequency (MAF) in the population within the ExAC database. The frequency for both non-Finnish Europeans (NFE) and all individuals (ALL) are shown.

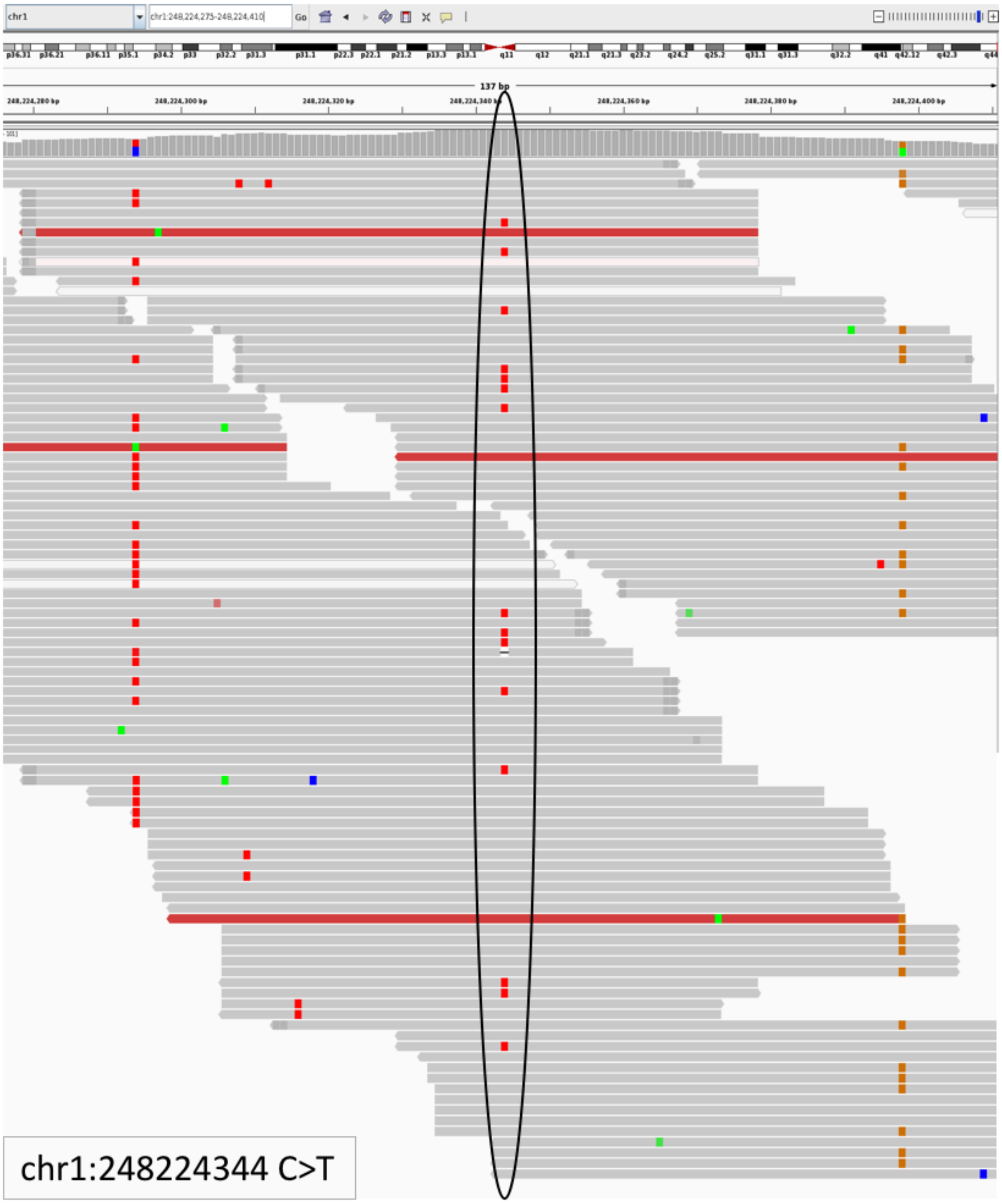
chr1
chr1:248,224,275-248,224,410
Go
137 bp
248,224,280 bp
248,224,300 bp
248,224,320 bp
248,224,360 bp
248,224,380 bp
248,224,400 bp
chr1:248224344 C>T

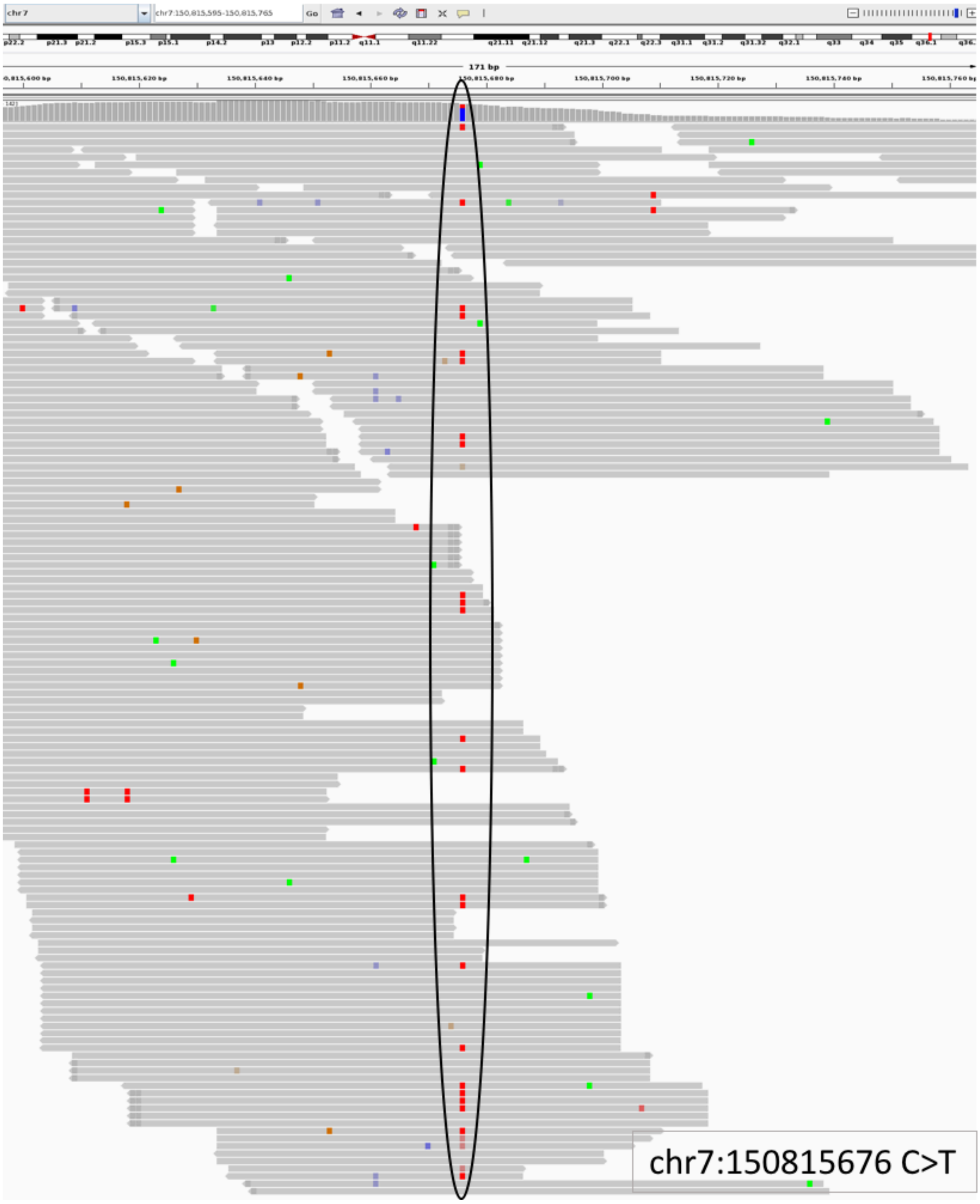
chr7:150815676 C>T

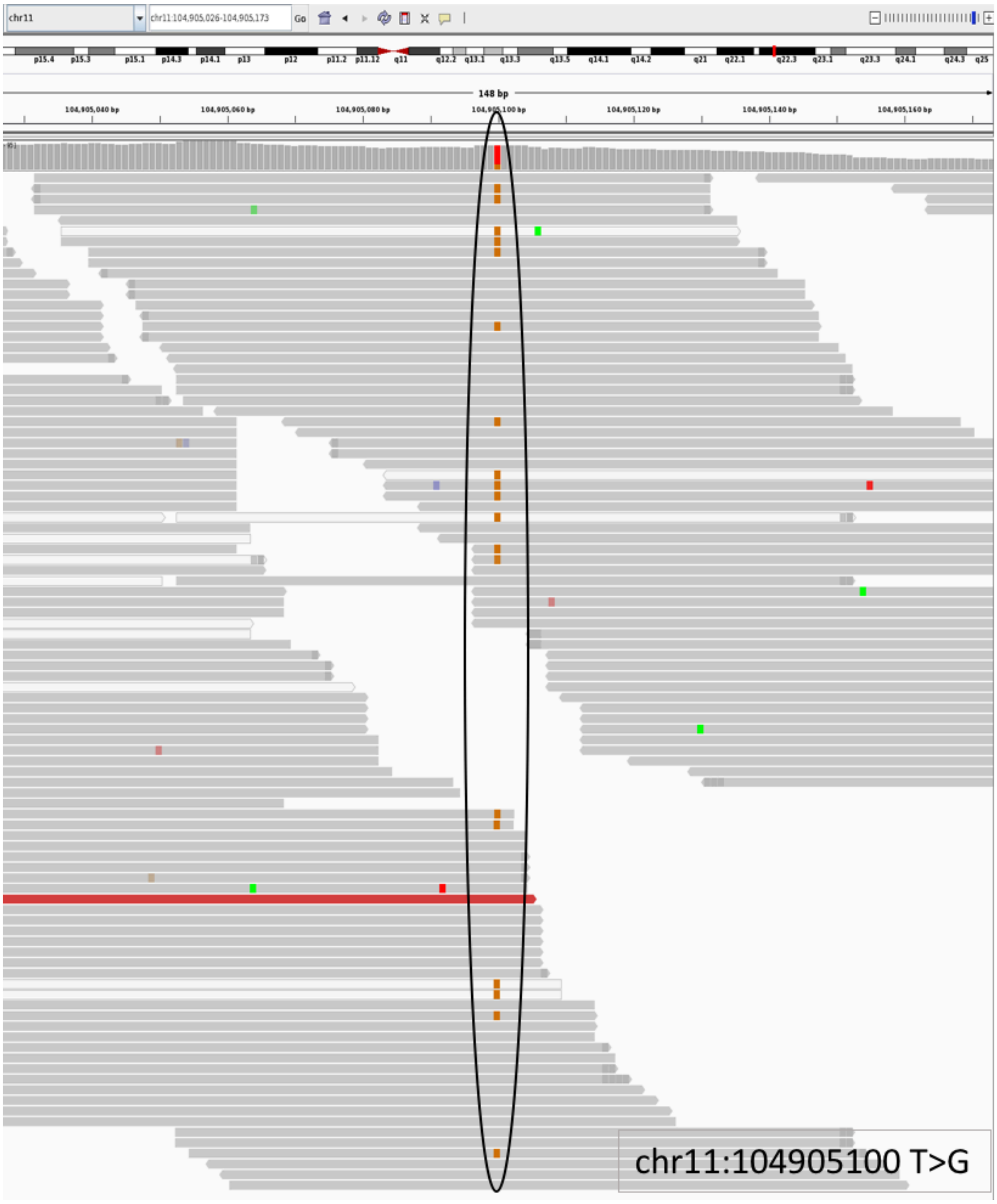
chr11
chr11:104,905,026-104,905,173
Go
148 bp
chr11:104905100 T>G

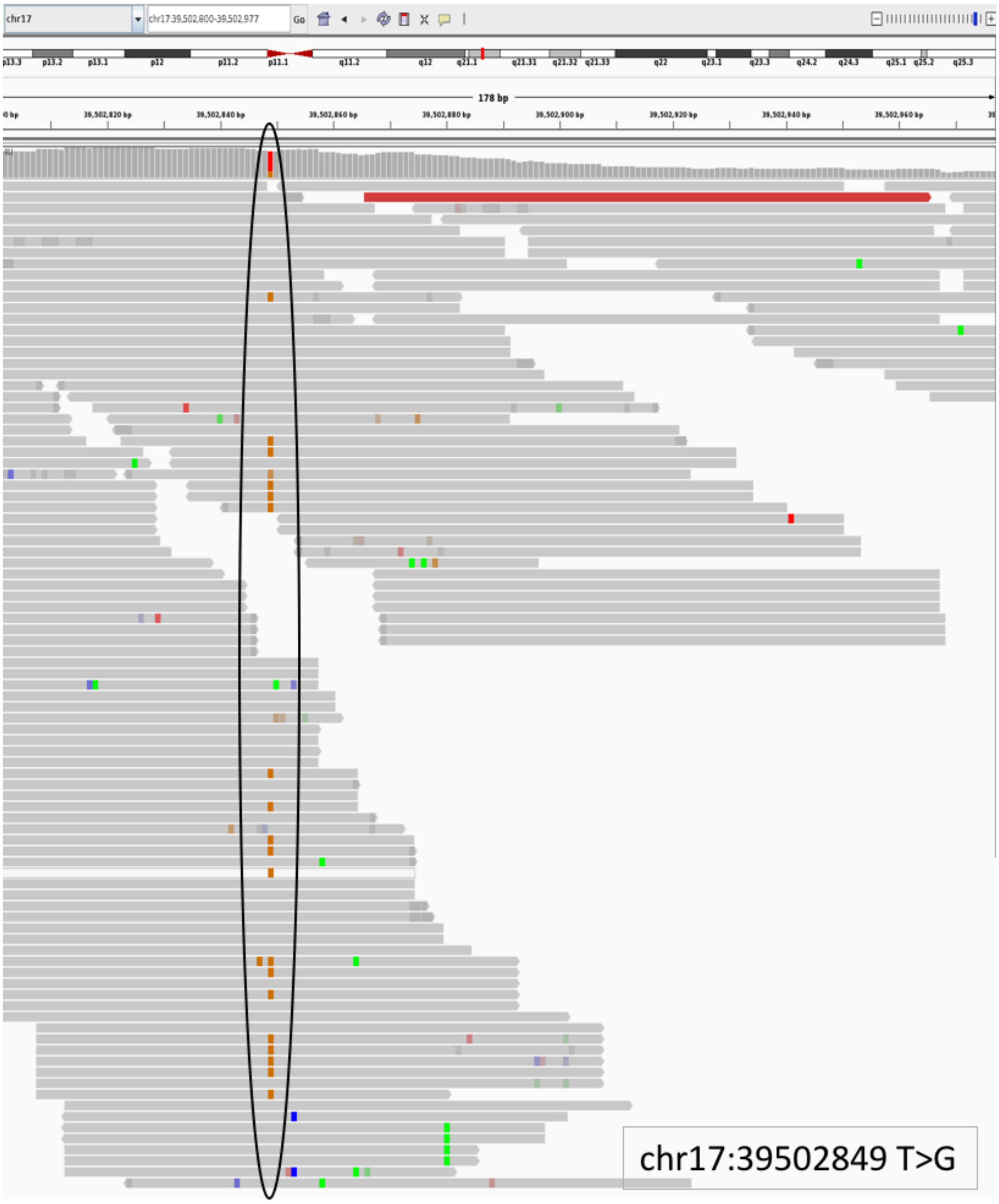
chr17
chr17:39,502,800-39,502,977
Go
178 bp
chr17:39502849 T>G

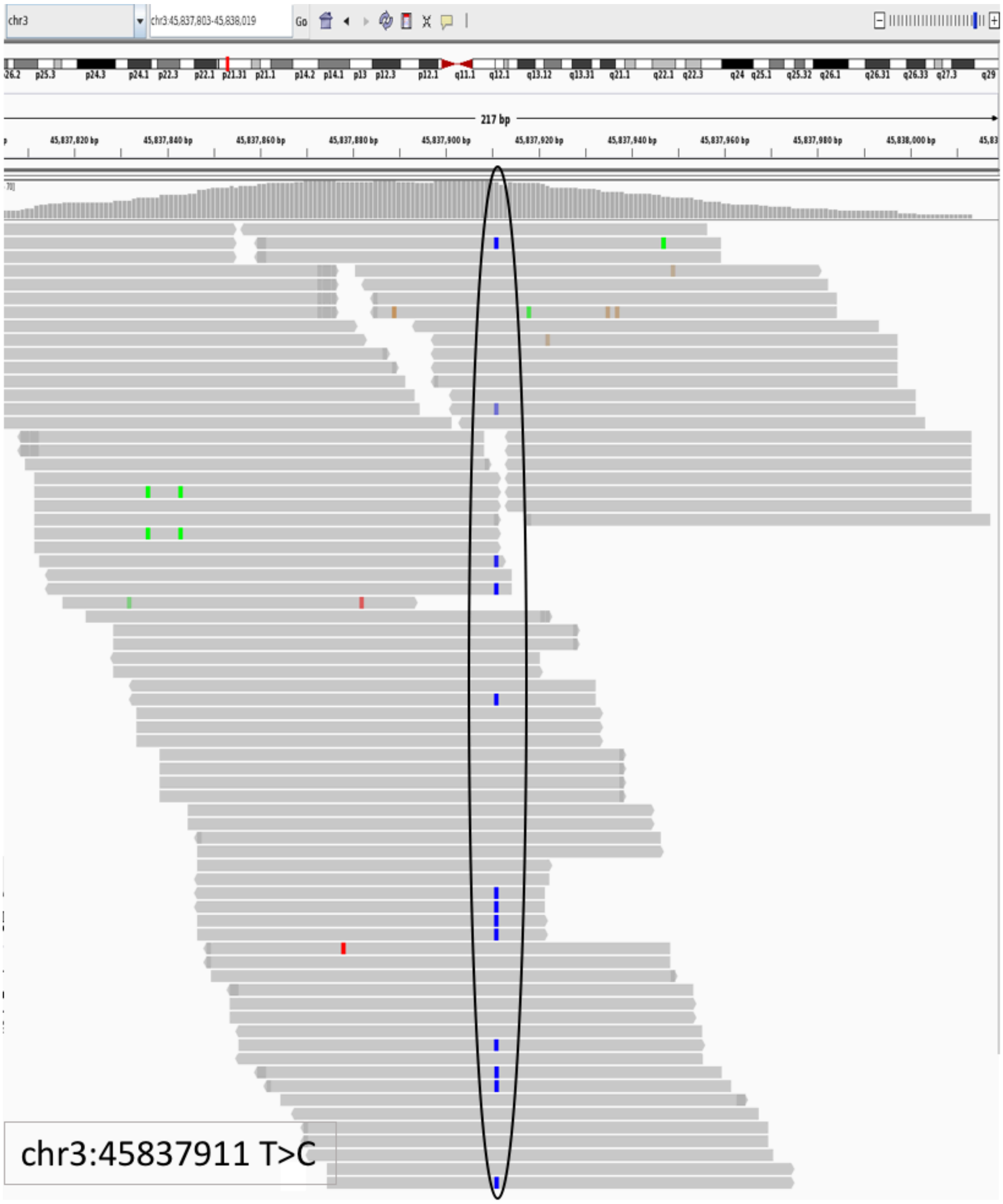
chr3
chr3:45,837,803-45,838,019
Go
217 bp
chr3:45837911 T>C

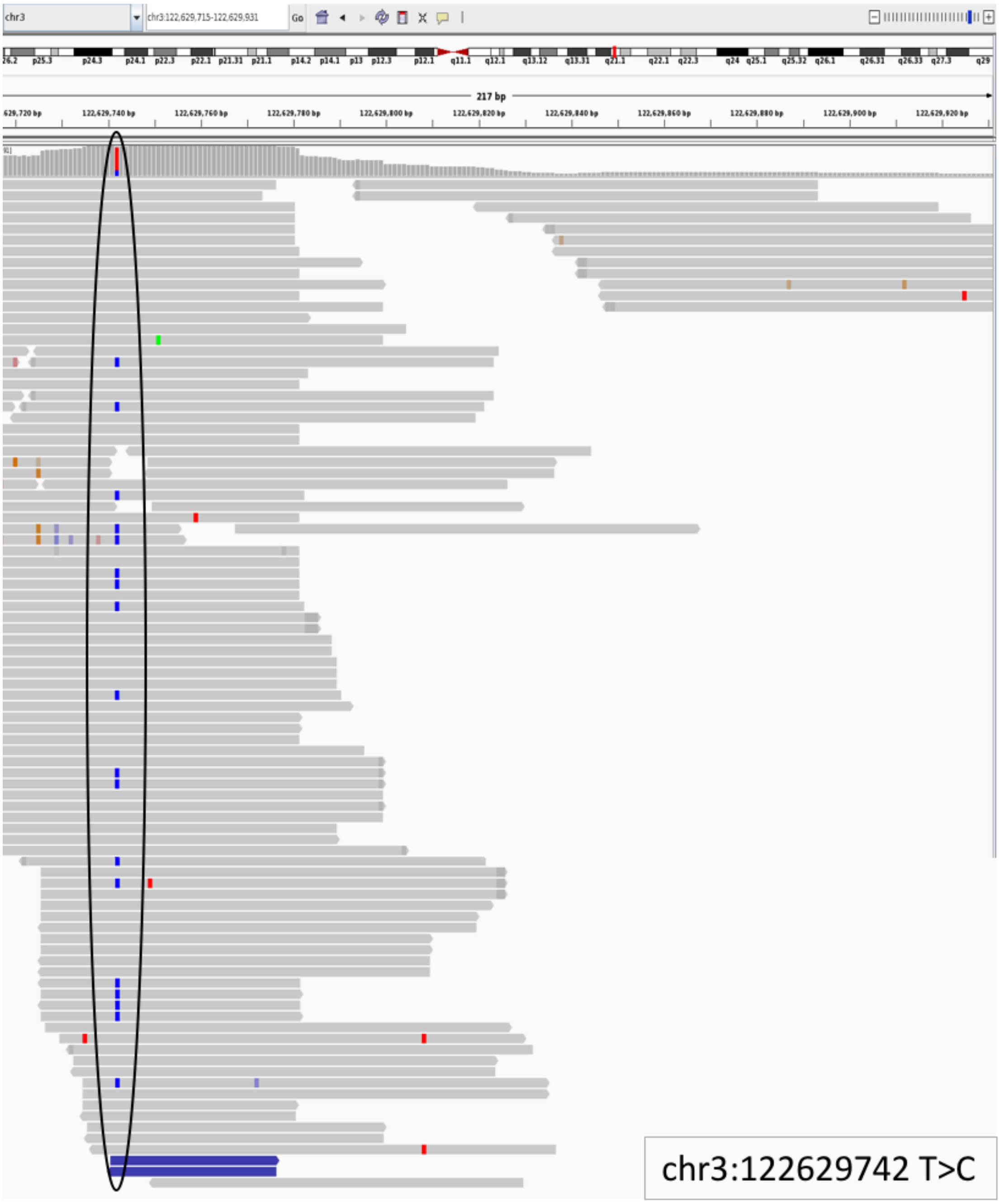
chr3
chr3:122,629,715-122,629,931
Go
217 bp
chr3:122629742 T>C

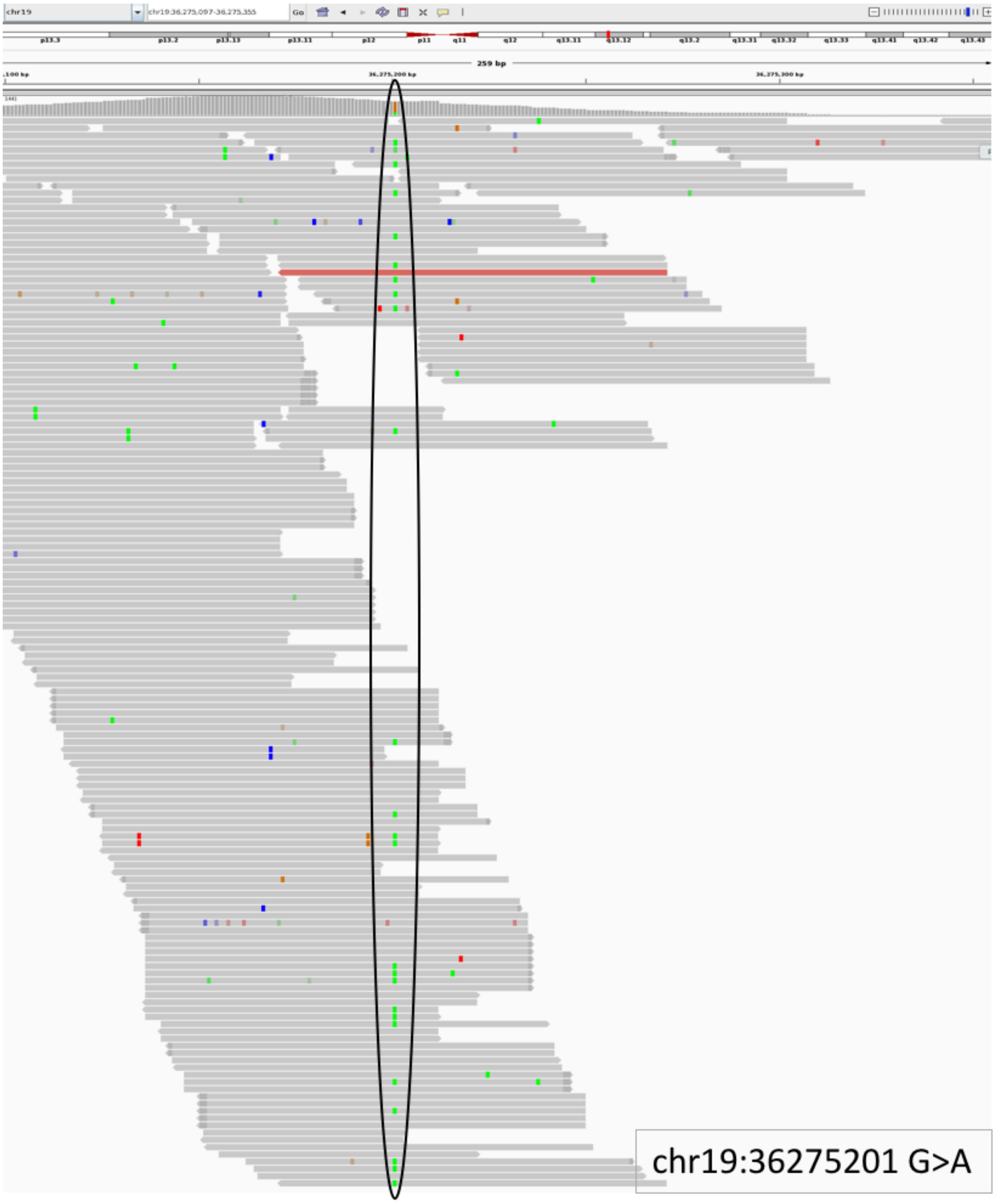
chr19:36275201 G>A

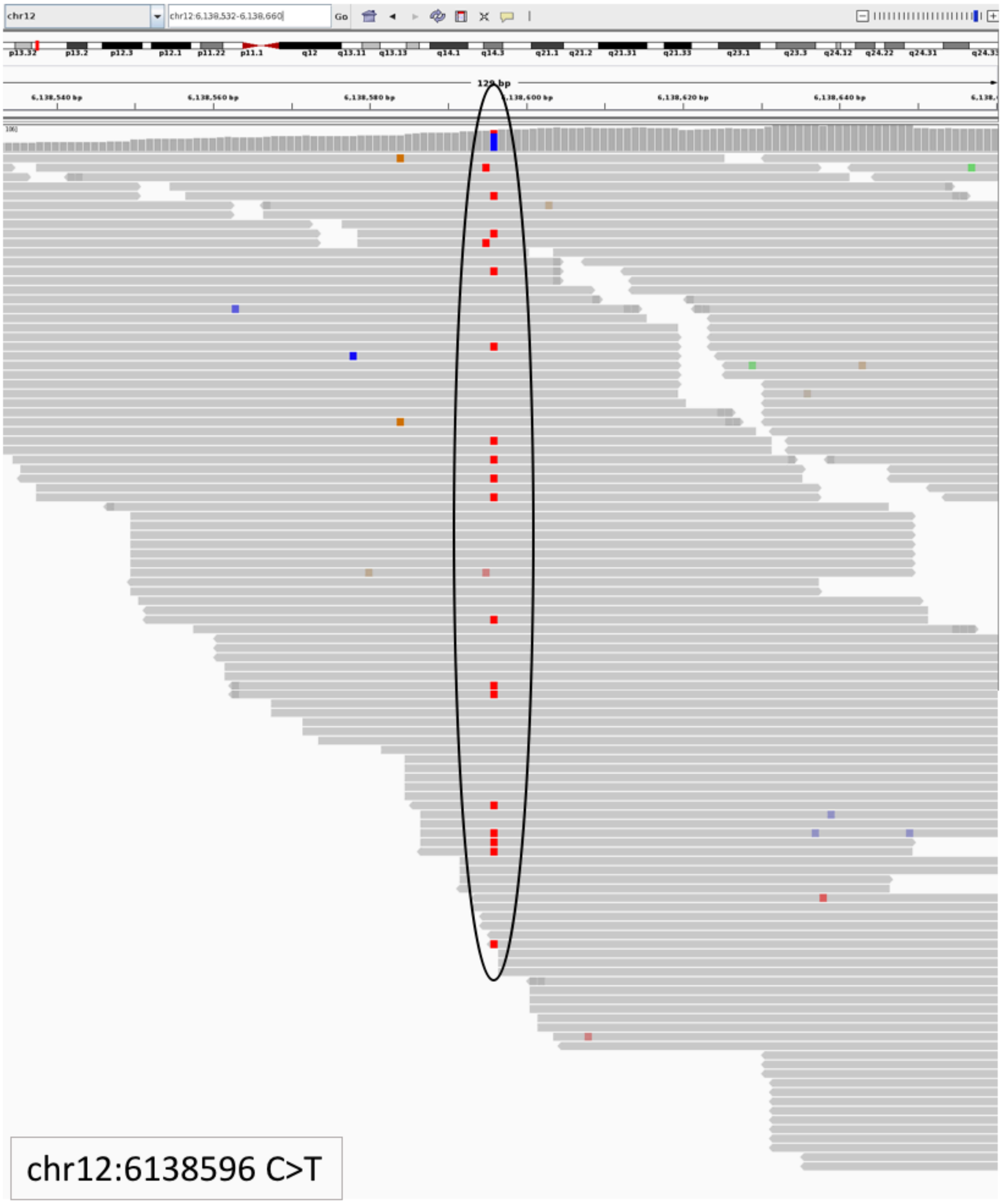
chr12
chr12:6,138,532-6,138,660
Go
129 bp
6,138,540 bp
6,138,560 bp
6,138,580 bp
6,138,620 bp
6,138,640 bp
chr12:6138596 C>T

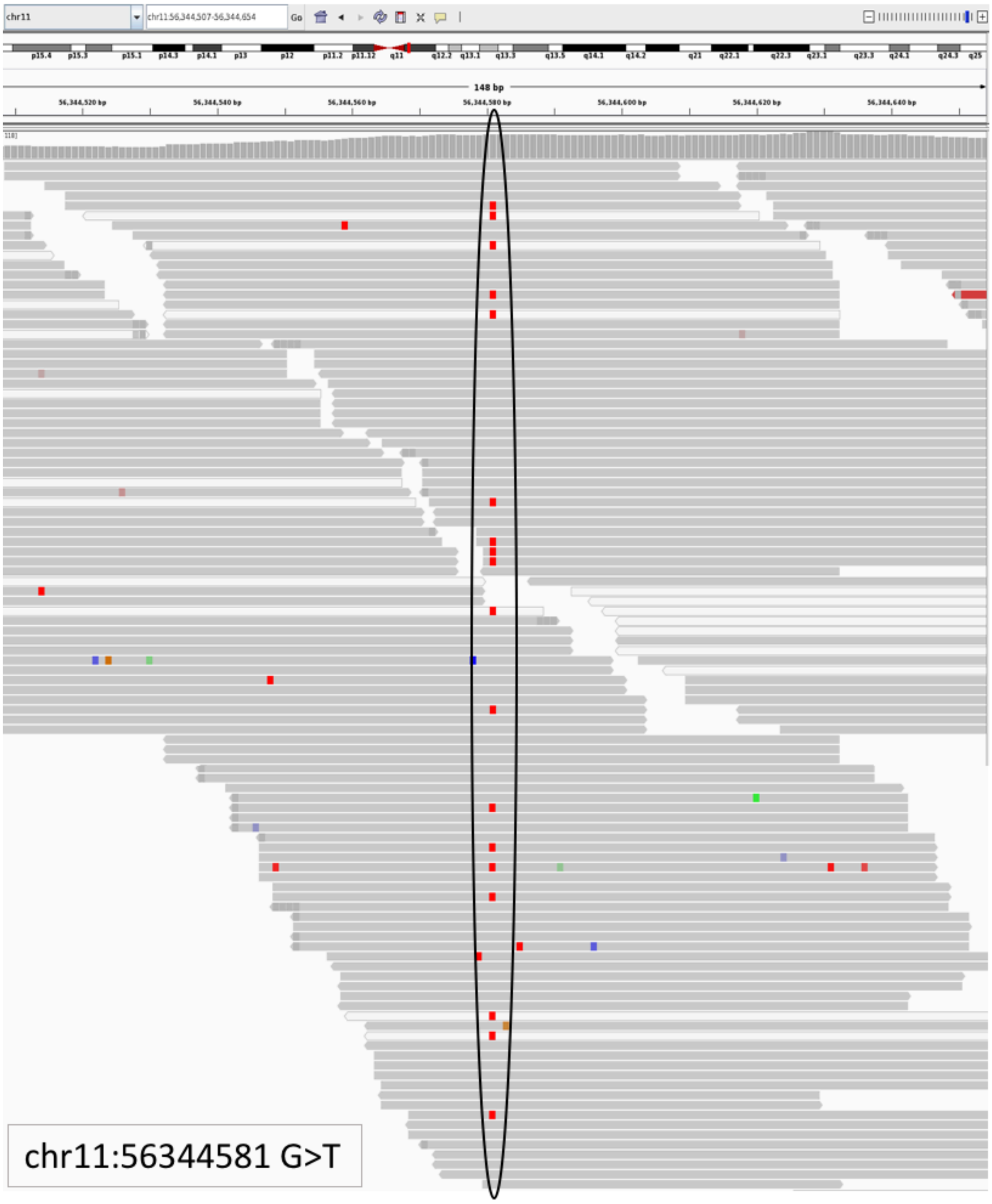
chr11
chr11:56,344,507-56,344,654
Go
148 bp
chr11:56344581 G>T

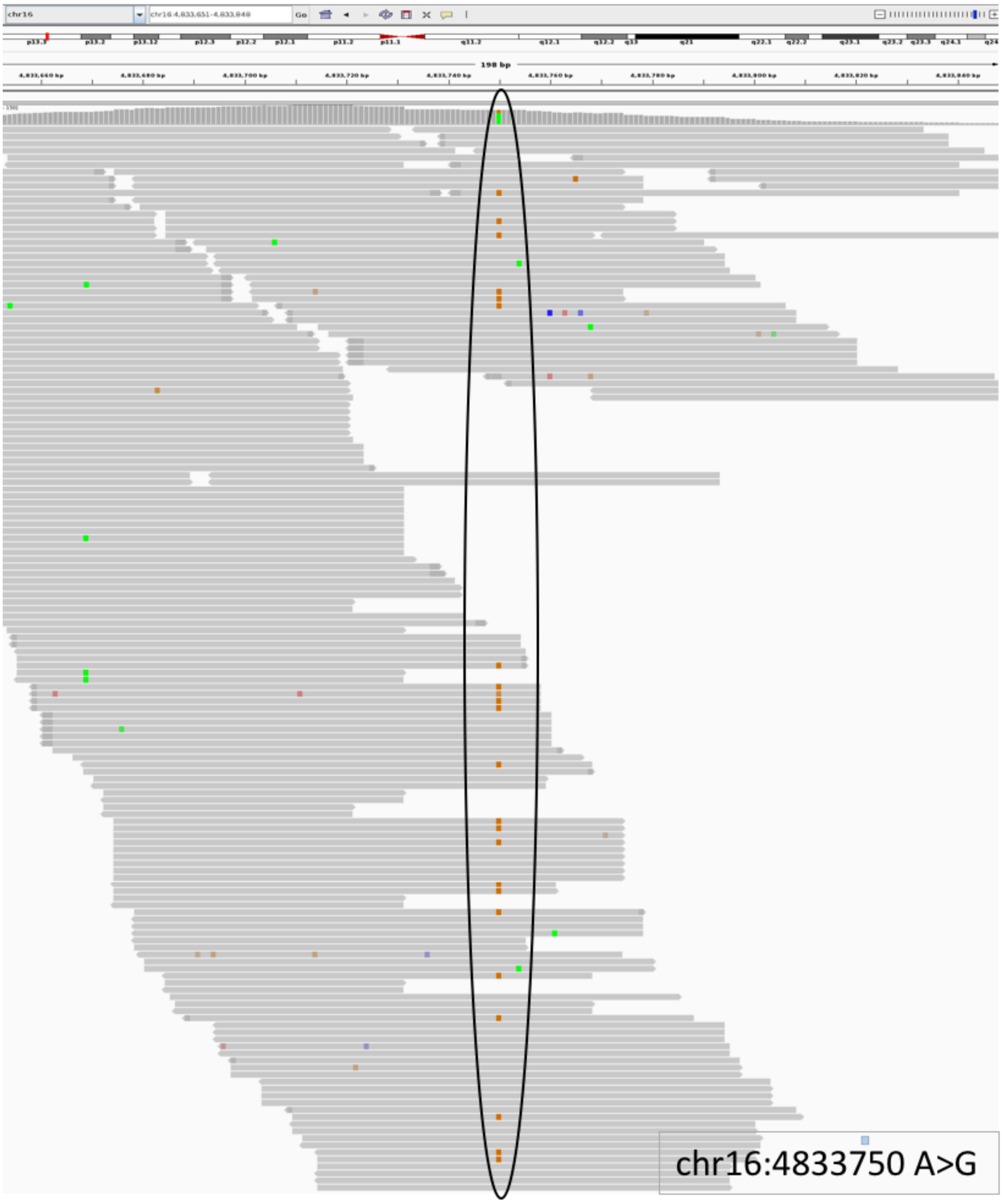
chr16:4833750 A>G

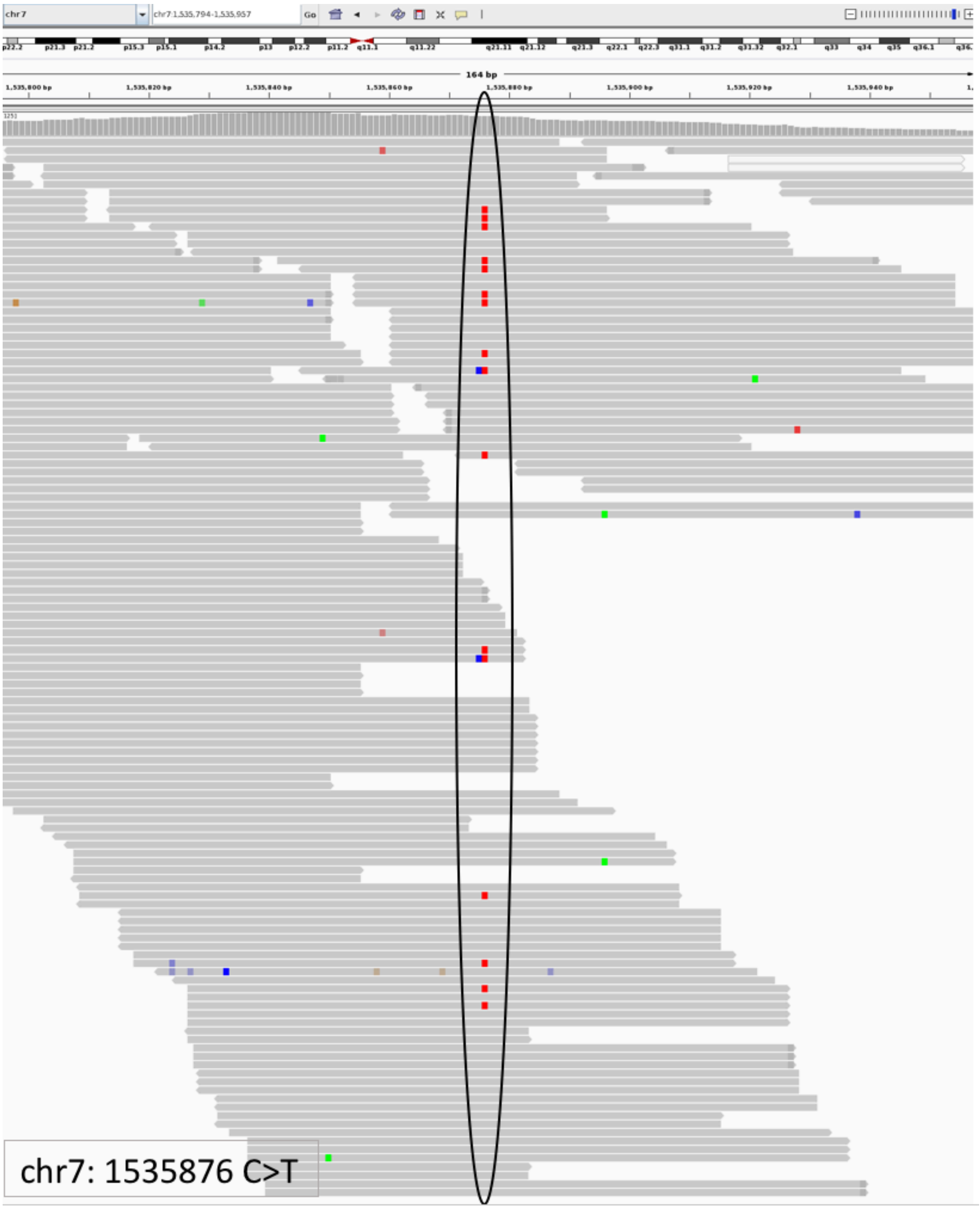
chr7: 1535876 C>T

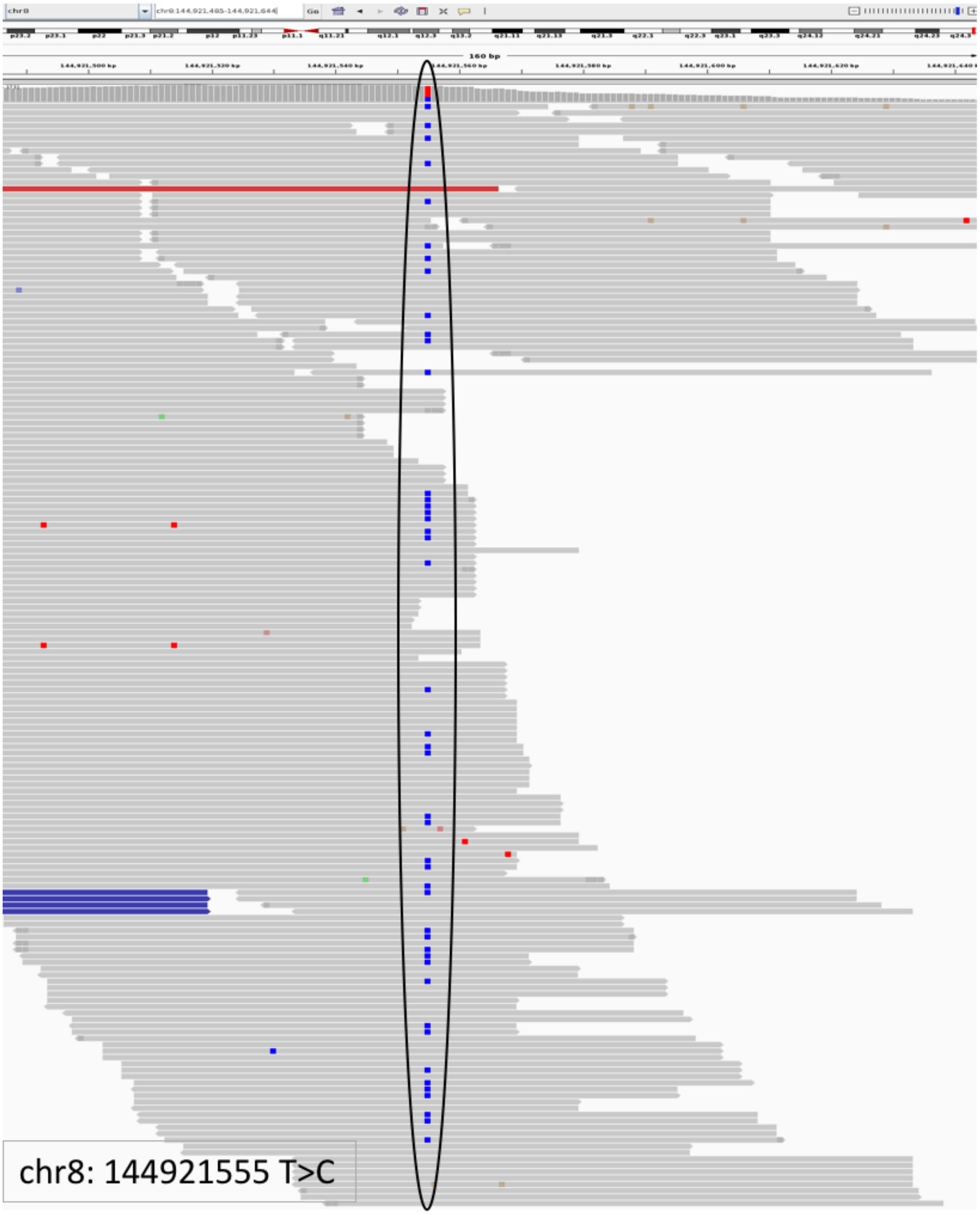
160 bp
chr8: 144921555 T>C

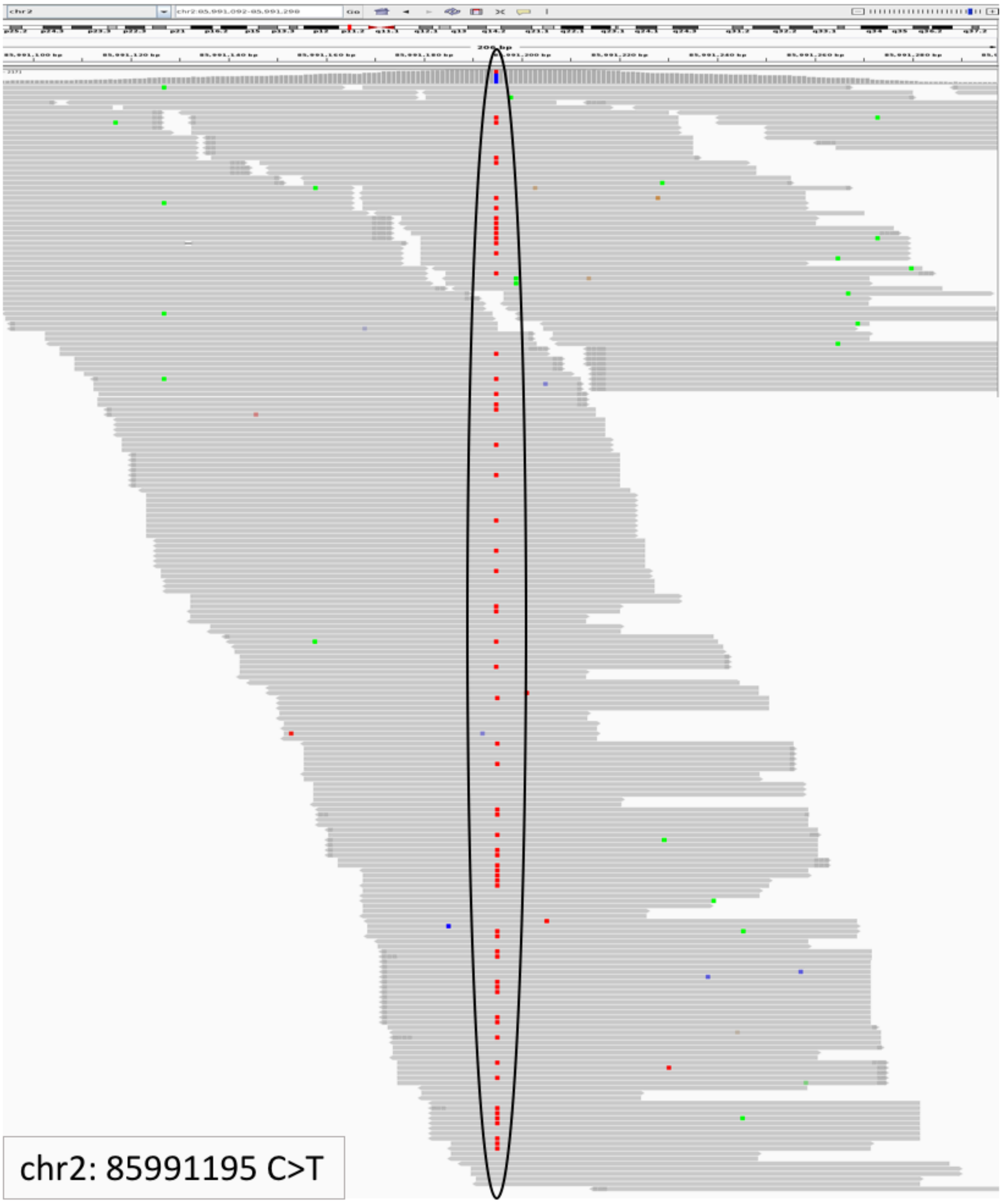

chr2: 85991195 C>T

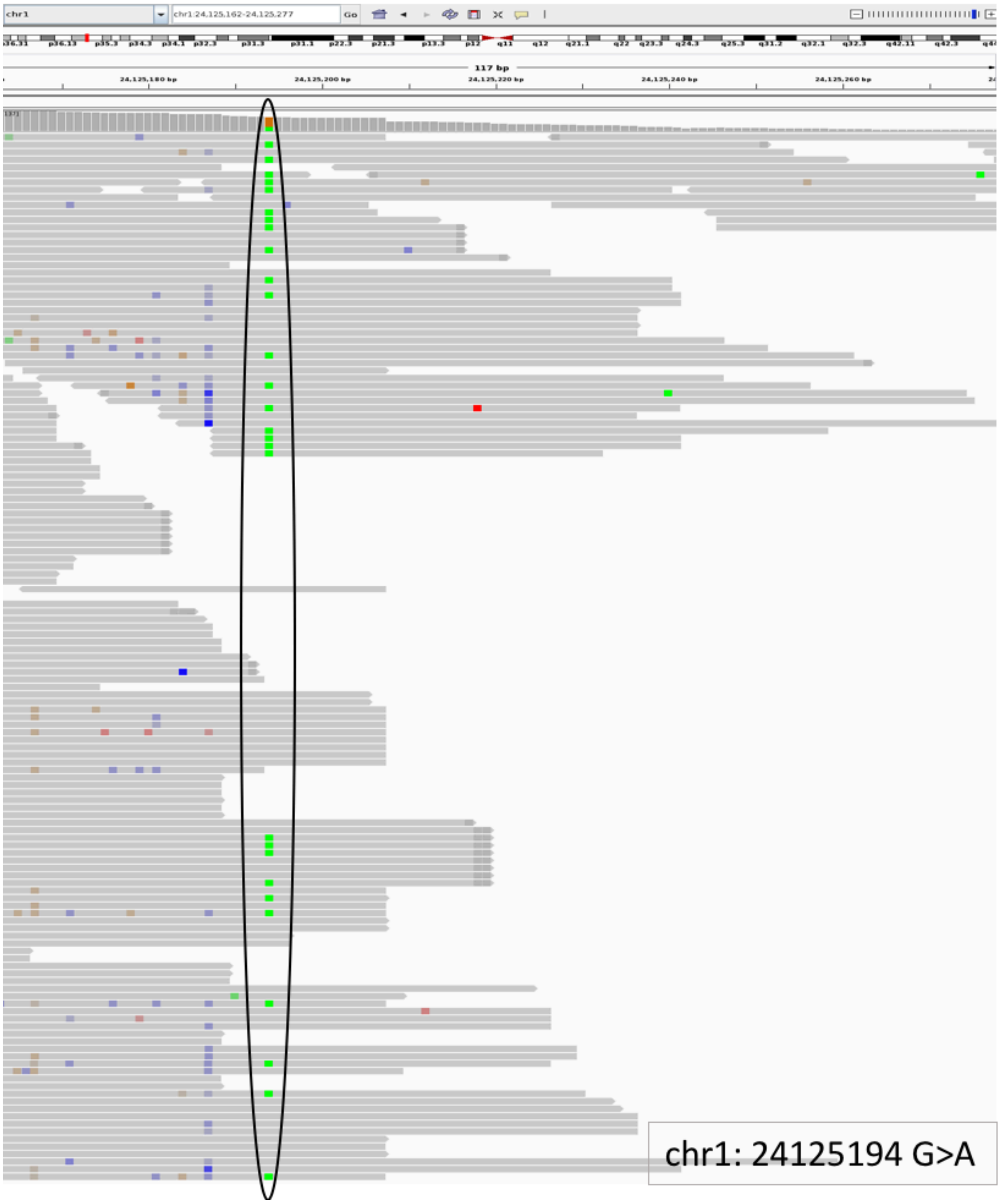
chr1: 24125194 G>A

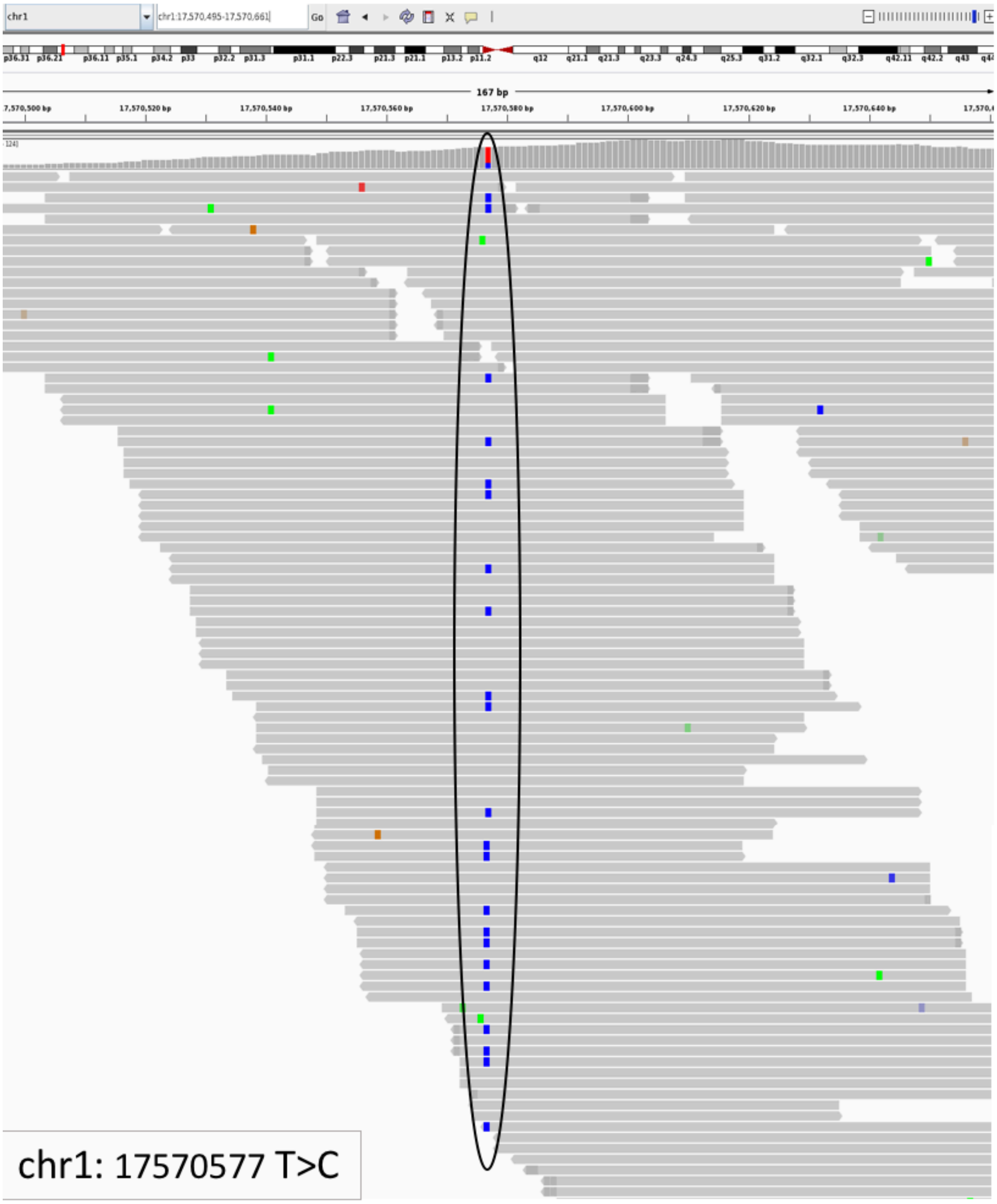
chr1: 17570577 T>C

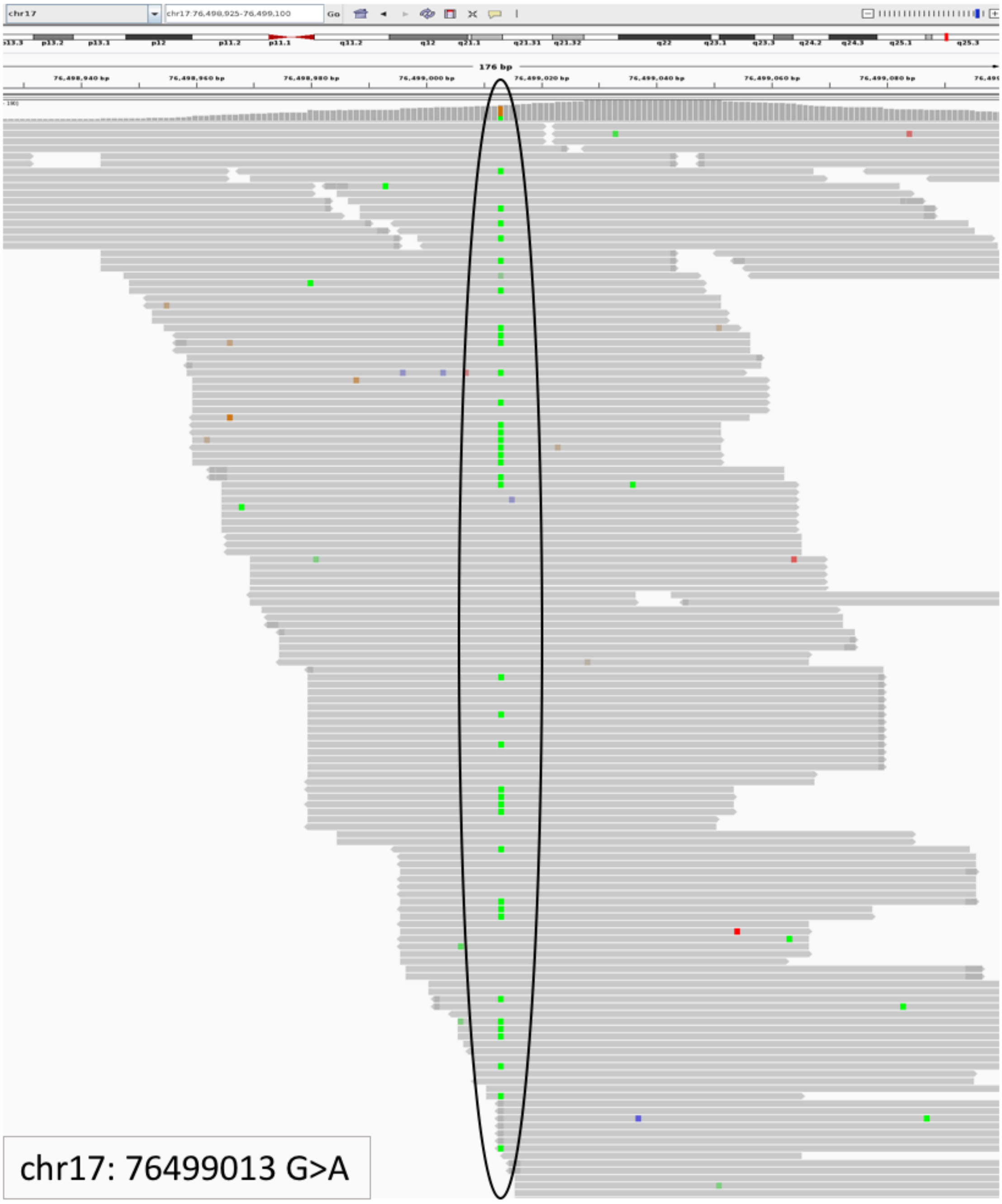
chr17
176 bp
chr17: 76499013 G>A

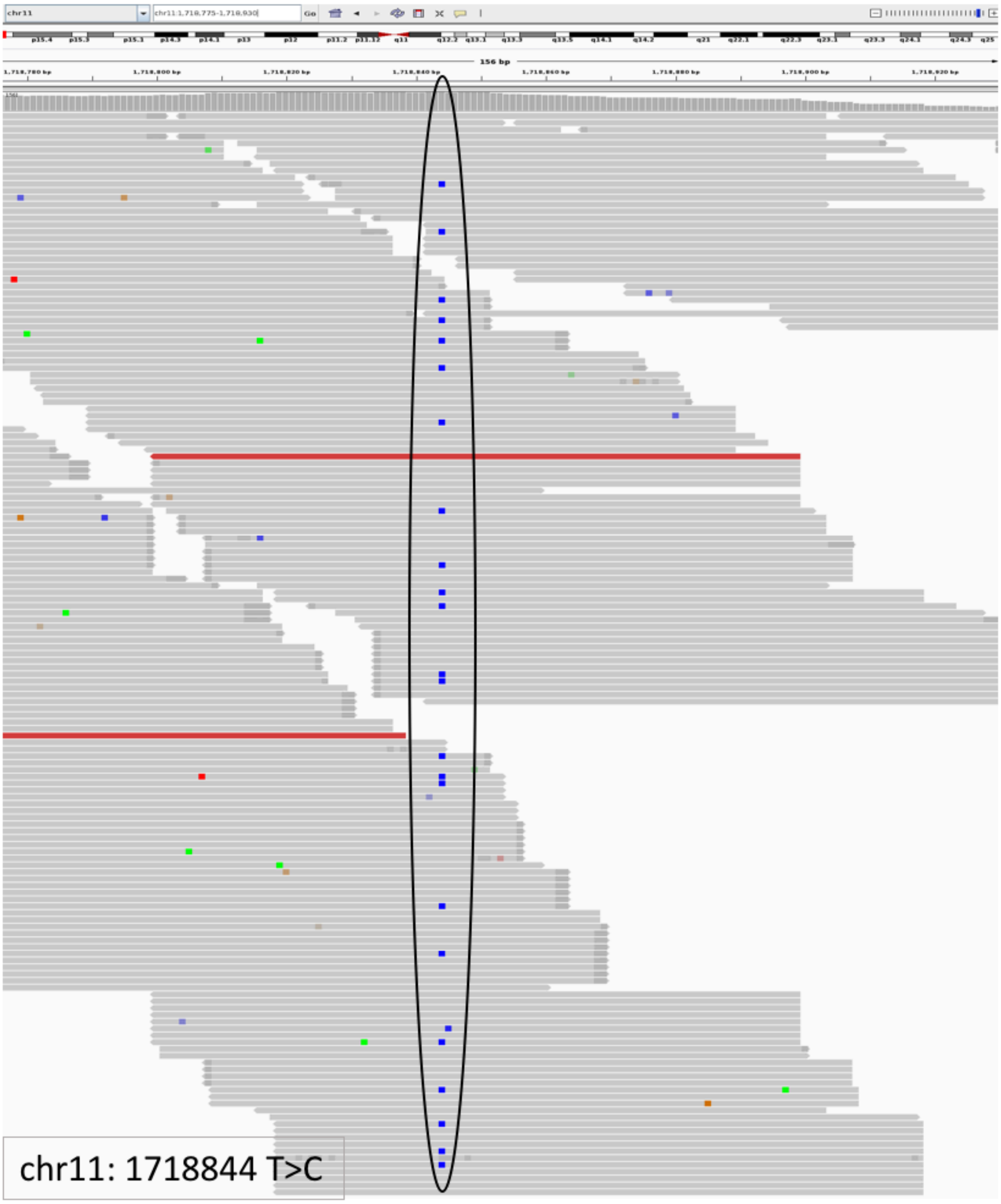

chr11: 1718844 T>C

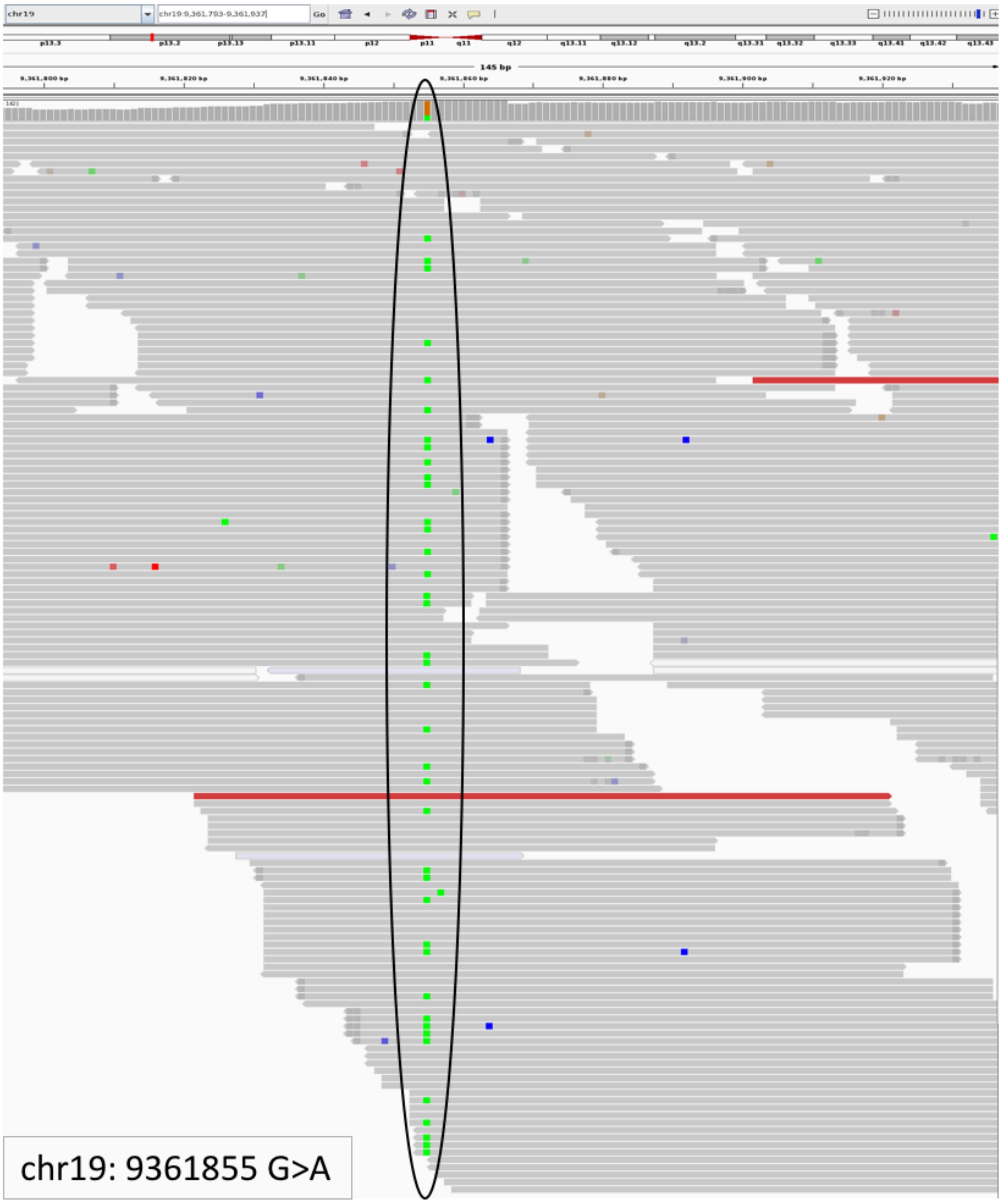
145 bp
chr19: 9361855 G>A

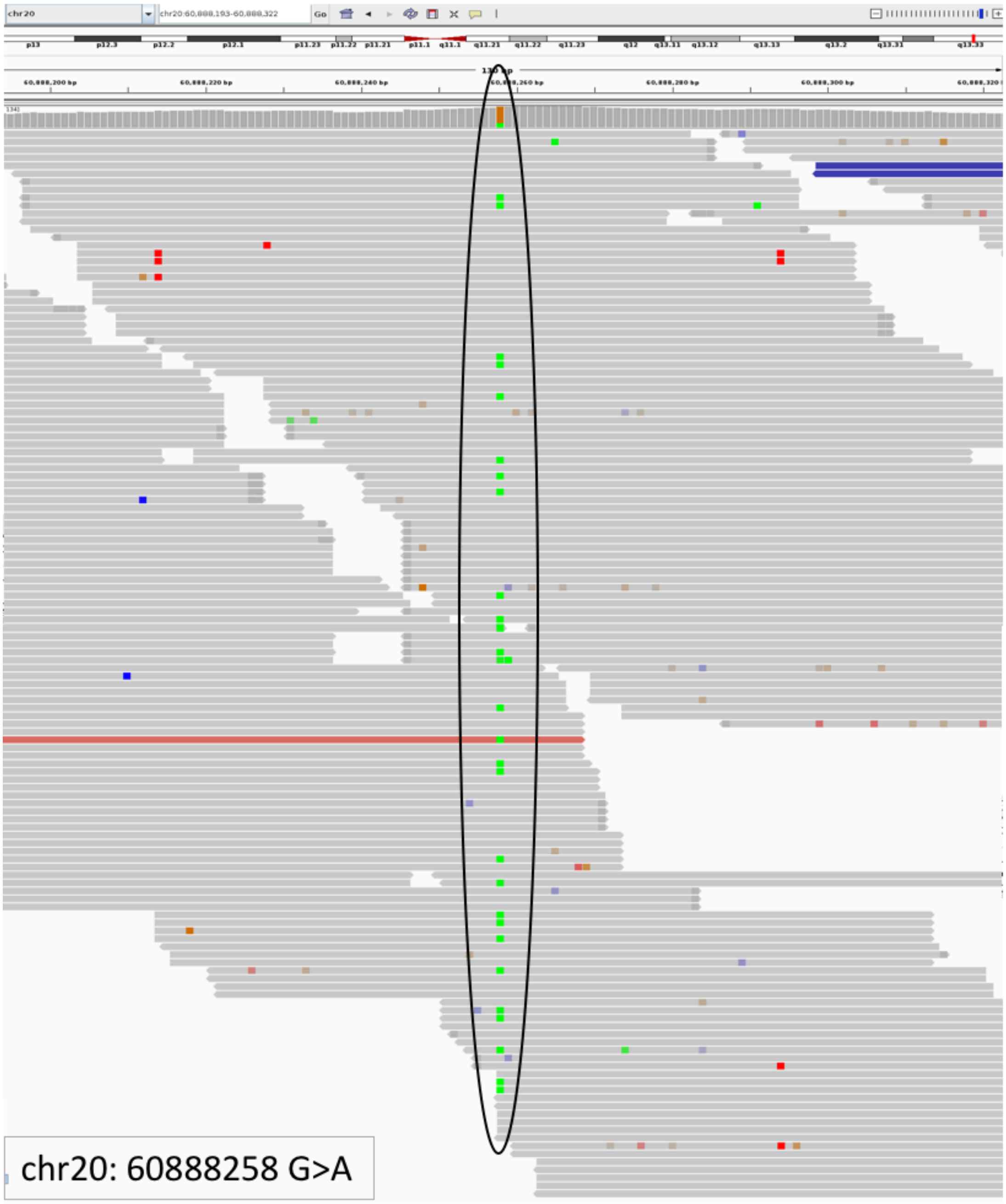

chr20
chr20:60,888,193-60,888,322
Go
130 bp
chr20: 60888258 G>A

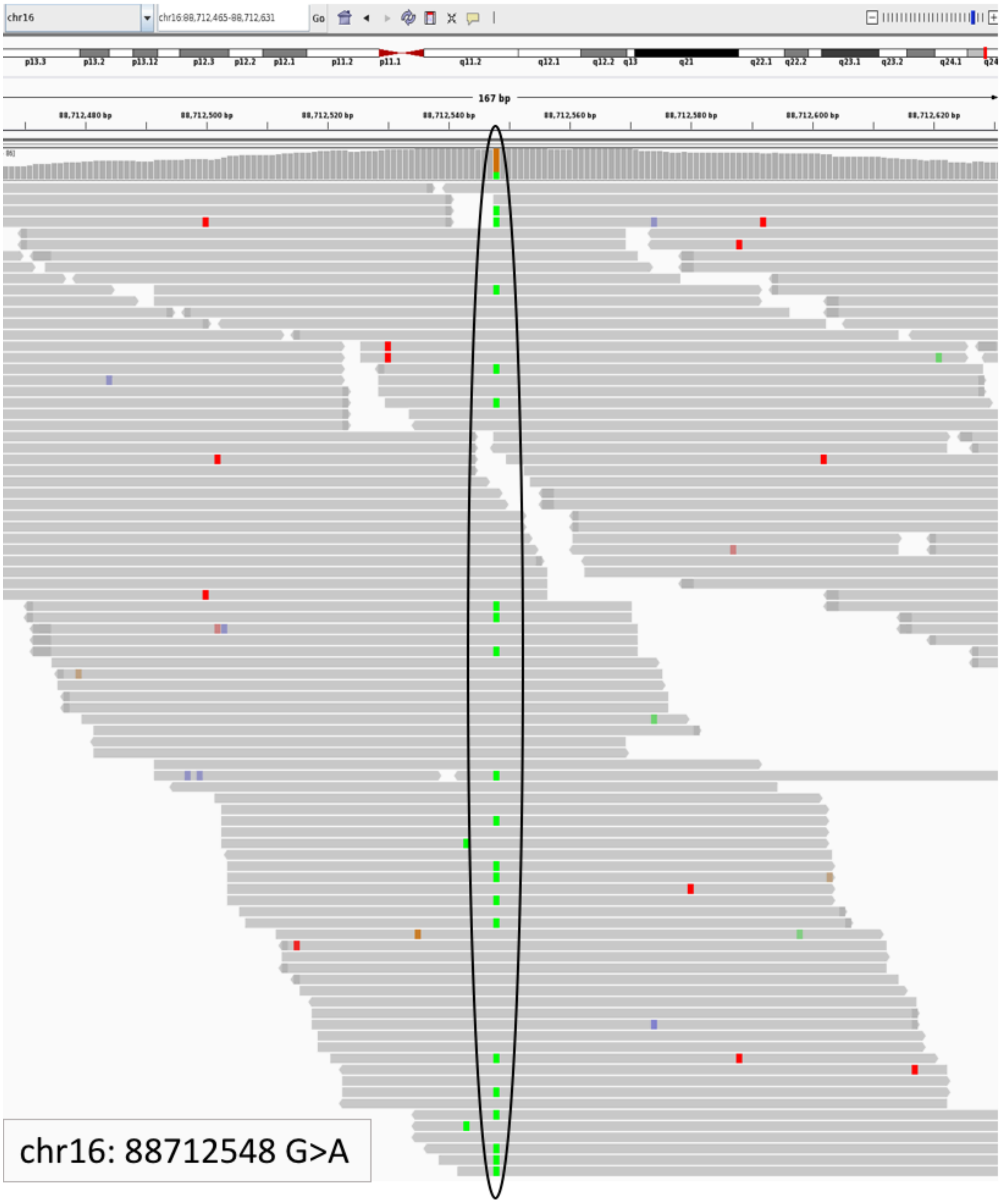
chr16
chr16:88,712,465-88,712,631
Go
167 bp
chr16: 88712548 G>A

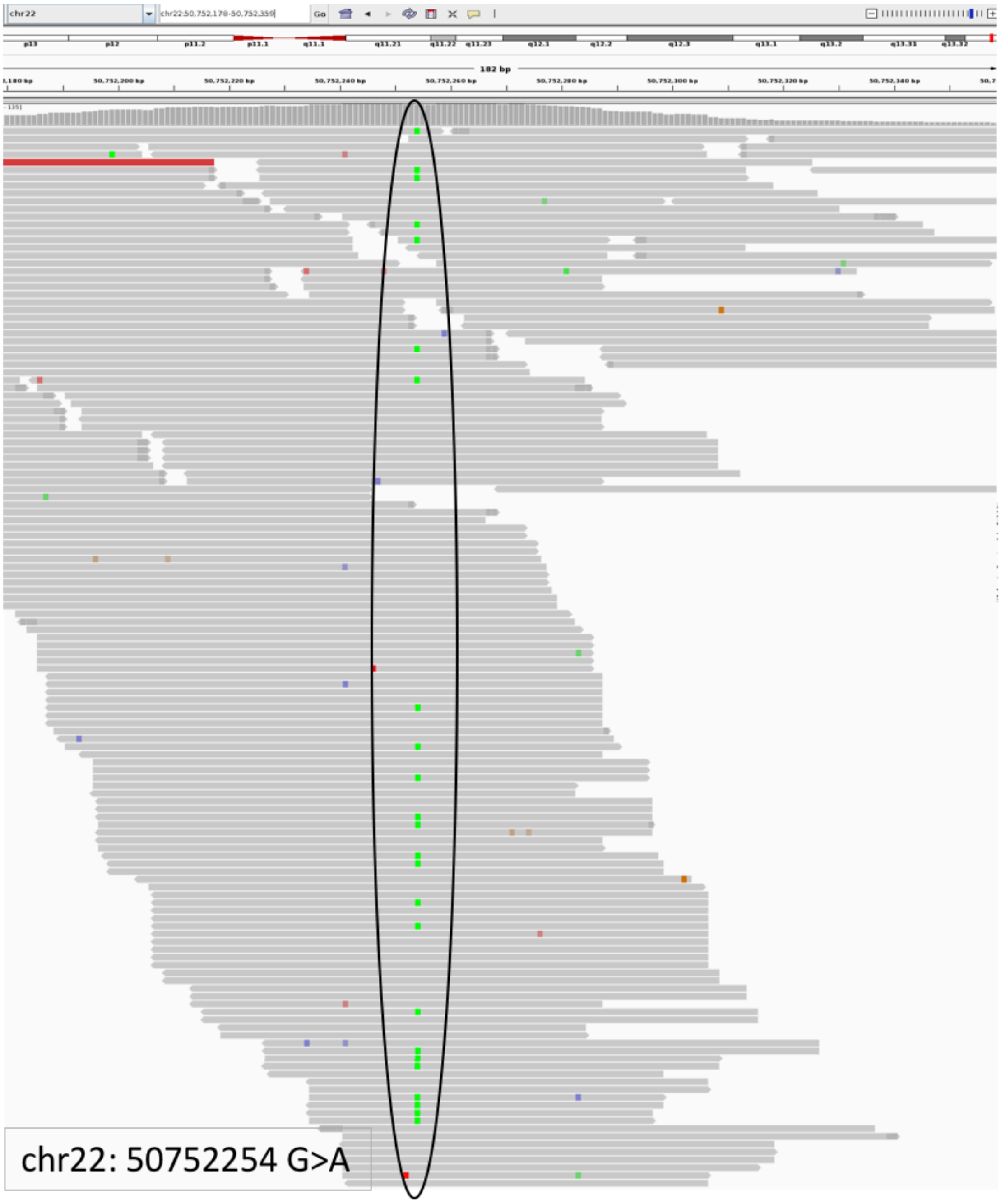
chr22
chr22:50,752,178-50,752,359
Go
182 bp
chr22: 50752254 G>A

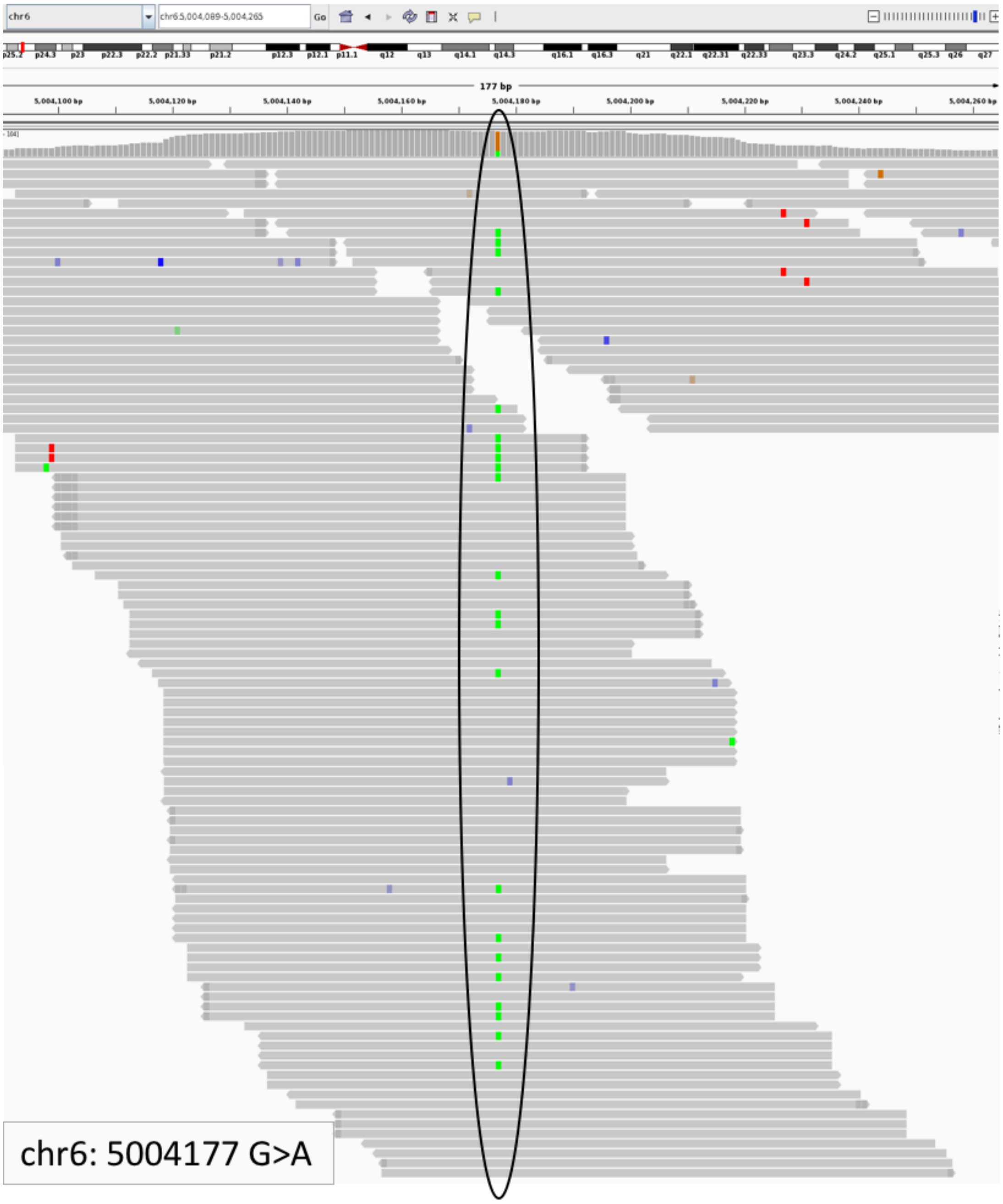

Supplementary Figure 4. The IGV browser images of validated somatic variants that were realigned in response to the anonymous reviewer. The position and allele change of each somatic variant is shown at the bottom of the image.
